# Nanoplasmonic Approaches for Sensitive Detection and Molecular Characterization of Extracellular Vesicles

**DOI:** 10.3389/fchem.2019.00279

**Published:** 2019-05-07

**Authors:** Tatu Rojalin, Brian Phong, Hanna J. Koster, Randy P. Carney

**Affiliations:** ^1^Department of Biochemistry and Molecular Medicine, University of California, Davis, Davis, CA, United States; ^2^Department of Biomedical Engineering, University of California, Davis, Davis, CA, United States

**Keywords:** exosomes, diagnostics, SERS, SPR, nanopillars, nanoarrays

## Abstract

All cells release a multitude of nanoscale extracellular vesicles (nEVs) into circulation, offering immense potential for new diagnostic strategies. Yet, clinical translation for nEVs remains a challenge due to their vast heterogeneity, our insufficient ability to isolate subpopulations, and the low frequency of disease-associated nEVs in biofluids. The growing field of nanoplasmonics is poised to address many of these challenges. Innovative materials engineering approaches based on exploiting nanoplasmonic phenomena, i.e., the unique interaction of light with nanoscale metallic materials, can achieve unrivaled sensitivity, offering real-time analysis and new modes of medical and biological imaging. We begin with an introduction into the basic structure and function of nEVs before critically reviewing recent studies utilizing nanoplasmonic platforms to detect and characterize nEVs. For the major techniques considered, surface plasmon resonance (SPR), localized SPR, and surface enhanced Raman spectroscopy (SERS), we introduce and summarize the background theory before reviewing the studies applied to nEVs. Along the way, we consider notable aspects, limitations, and considerations needed to apply plasmonic technologies to nEV detection and analysis.

## Introduction

Nanoscale extracellular vesicles (nEVs) encompass a heterogeneous grouping of naturally occurring nanoparticles that are endogenously secreted by all cells tested to date (Mathieu et al., 2019). As researchers have begun to unravel the structure and function of these lipid-bilayer wrapped nanoscale assemblies, numerous analytical technologies have been applied to investigate nEVs in the context of disease detection and diagnosis (Coumans et al., 2017; Théry et al., 2018). No category of techniques may have more promise than nanoplasmonics, the field of engineering nanoscale metallic surfaces for the significant enhancement of analytical signals, both in magnitude and also in terms of molecular specificity. While promising, nanoplasmonic innovations are difficult to translate into clinical diagnostic platforms due to both the inherent complexity of the techniques themselves, but also as a result of the compositional and temporal heterogeneity of biological agents inside the human body during disease progression. Such heterogeneity is a particular hallmark of nEVs (Tkach et al., 2018).

The motivation of this review is to comprehensively describe the state of the art in plasmonics sensing of nEVs in the context of disease detection and monitoring. We begin with an overview of nEV structure and function before assessing the work being performed at the intersection of nanoplasmonics-based nEV detection. We finish with a critical overview of the current positioning of the field. Throughout, we emphasize the challenges in enriching and analyzing nEVs and carefully consider the limitations of each presented methodology.

### nEV Background and Characteristics

The first descriptions of nEVs involved careful transmission electron microscopy (TEM) analyses of maturing reticulocytes, cells that specialize in recycling a large portion of their contents, and nEVs spent much of the last 30 years associated primarily with their function in secreting cellular waste (Johnstone et al., 1987; Raposo et al., 1996). In the early 2000s, a handful of researchers began to demonstrate the immune-stimulating effects of nEVs *in vivo* (Théry et al., 2002). A notable sea-change soon arose, when it was reported that isolated nEVs contained, and were capable of delivering, functional RNAs, establishing a strong physiological relevance (Valadi et al., 2007). Since this paradigm shift, the field of nEVs has experienced an exponential growth in published reports describing their structure and function. Although our understanding continues to rapidly evolve, it is now well-accepted that nEVs are a highly diverse and complex group of nanoparticles exhibiting vast biomolecular heterogeneity and likely contributing to numerous functions throughout all biological kingdoms, capable of acting both in their local environment and also released in circulation (Yáñez-Mo et al., 2015).

Various types of nEVs and their contents have been implicated in controlling, or at least found to be associated with, numerous homeostatic processes, including cell viability and proliferation, cellular differentiation, immunosuppression, bone formation, modulation of blood pressure, and both promotion and suppression of angiogenesis (Kusuma et al., 2018). They are reported to modulate extracellular matrix remodeling to promote cell intravasation and migration via trafficking of numerous matrix-remodeling enzymes (Nawaz et al., 2018). Many of their physiological claims are also associated with pathological conditions, implicating nEVs as mediators of a host of cardiovascular and metabolic diseases (Koenen and Aikawa, 2018), arthritis and inflammatory disease (Buzas et al., 2014), neurological disorders (Coleman and Hill, 2015; Janas et al., 2016), and cancers. Much effort has been applied to teasing out the position of nEVs in cancer pathology, namely their role in pre-metastatic niche formation (Costa-Silva et al., 2015) via integrin-mediated organotropic targeting (Hoshino et al., 2015) and followed by attracting/repelling certain populations of immune cells (Bobrie and Théry, 2013), stimulating angiogenesis (Aguado et al., 2017), matrix remodeling (Nawaz et al., 2018), and reprogramming of target cell transcriptomes to promote tumorigenesis (Melo et al., 2014). The role of nEVs in host-pathogen communication is also a prevalent topic (Deatherage and Cookson, 2012; Dauros Singorenko et al., 2017). Accumulating evidence demonstrates that virus-modified or virus-containing nEVs contribute to spread and immune evasion, particularly in the context of promoting cancer (Meckes, 2015).

Many therapeutic claims for nEVs have been introduced, for example nEVs have been proposed for use in cancer vaccination (Tan et al., 2010), resistance to viral infection (Gould et al., 2011), and to challenge demyelinating diseases (Osorio-Querejeta et al., 2018). Released from mesenchymal stromal cells (MSCs), nEVs are largely considered as potent cell-free regenerative agents, found to be effective in bone and tissue repair, and as protective or curative agents in ischemia, sepsis, renal fibrosis, and osteopenia (Jing et al., 2018). MSC-nEVs are immune privileged and can be loaded with exogenous therapeutic agents, therefore there is much interest in their use as targeted drug delivery agents (Barile and Vassalli, 2017). While technical issues have presently prevented scalable methods to do so effectively, several promising clinical trials are currently underway (Wilson et al., 2018).

### Structural and Molecular Heterogeneity of nEV Subpopulations

**Figure 1:**
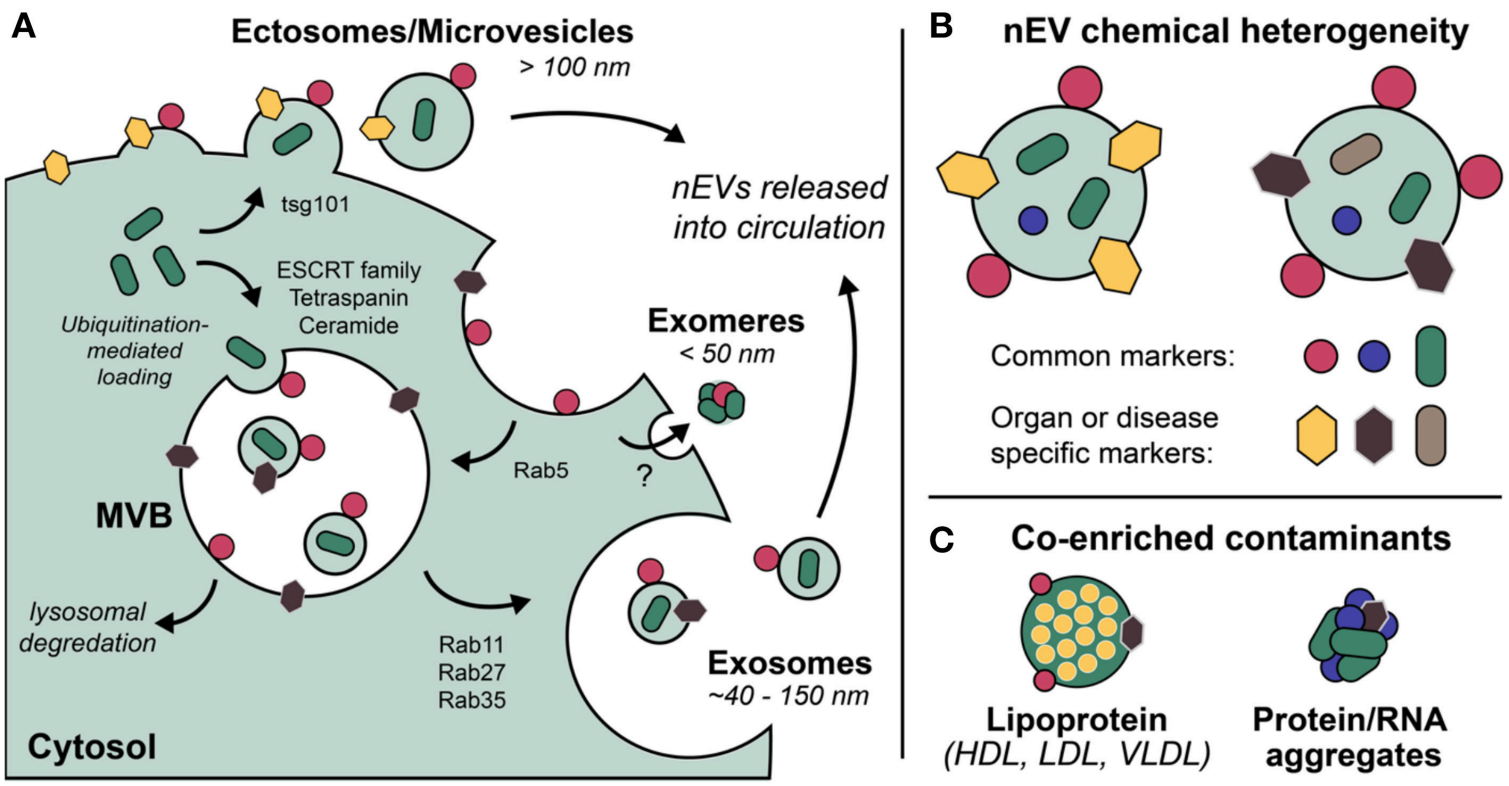
Extracellular vesicles (EVs) are a grouping of heterogeneous nanoscale assemblies of **(A)** various cellular biogenetic origin including but not limited to MVB-dependent release (exosomes) and direct budding (ectosomes, MVs), **(B)** exhibiting differential chemical heterogeneity depending on organ or disease specific context. **(C)** Lipoprotein particles, ribonucleoproteins, and protein aggregates are often co-enriched with EVs as a result of their overlapping physicochemical properties, i.e., nominal size and density.

Exosomes are the most well-defined and well-studied nEV subtype, although the term has come to mean different things (Gould and Raposo, 2013). Most appropriate may be the biogenetic usage of the term, i.e., those vesicles originating as ESCRT-dependent invaginations of early endosomes that are released into circulation upon fusing of the resultant multi-vesicular bodies (MVBs) with the plasma membrane (Figure 1). Importantly, if the Rab GTPases that modulate MVB/plasma membrane fusion during the release of exosomes are knocked down, cells still excrete nEVs, suggesting alternative methods of vesicle formation and release (Ostrowski et al., 2010; Cocucci and Meldolesi, 2015; Blanc and Vidal, 2017). These may include ESCRT-independent, ceramide-based mechanisms and direct budding (so-called ectosomes) (Trajkovic et al., 2008; Cocucci and Meldolesi, 2015). Exomeres, a new class of circulating nanoparticle, were recently defined as <50 nm aggregates composed of lipid, protein, and nucleic acid but without a defined lipid bilayer, nor reflecting classic lipoprotein composition (Zhang H, et al., 2018). Larger EVs have also been described, with one population termed large oncosomes (LOs) found to be enriched for large fragments of chromosomal DNA, challenging previously held notions that DNA is trafficked in smaller nEVs (Vagner et al., 2018). While this review makes the distinction of describing *nanoscale* EVs, also prevalent in blood circulation are larger microvesicles (MVs) and apoptotic bodies (both of which bleb directly from the cell membrane) that are often co-enriched during standard isolation preps (Sódar et al., 2016). Moreover, lipoprotein particles (LPPs) and ribonucleoproteins (RNPs) are also relevant traffickers of biomolecules, particularly extracellular RNAs (Wei et al., 2017), and also contaminate nEV preps. Methods capable of distinguishing the various nanoscale vehicles are badly needed (Figure 1).

In general, size is a major driver of nEV heterogeneity, though it remains difficult to discretely separate vesicles according to size (Zijlstra and Di Vizio, 2018). Quoted nominal dimensions for exosomes and related nEV subclasses are numerous, though trend toward a range of ~40–150 nm (Coumans et al., 2017), with MVs/LOs found up to 10 μm in diameter (Vagner et al., 2018). It is recognized that isolation/enrichment methods, cryopreservation storage (e.g., time, temperature, freeze-thaw cycling), and analytical approaches all bias measured size range, morphology, and degree of aggregation (Kusuma et al., 2018). Contradictory trends for evolution of nEV size distributions at various storage conditions persist (Jeyaram and Jay, 2018), thus working with freshly isolated vesicles is preferred when possible. Some alternatives such as lyophilization or incorporation of additives to improve stability of banked nEVs have been introduced (Kusuma et al., 2018).

Numerous distinctive classes of biomolecules make up nEVs, including lipids, coding and small non-coding nucleic acids, proteins and biologically active peptides, carbohydrates, hormones, growth factors, and structural components like fibronectin and actin (Yáñez-Mo et al., 2015). The extent of glycosylation of nEVs plays a role in trafficking and function, in addition to acting as a handle for therapeutic and diagnostic application (Williams et al., 2018). The cargo and the source of nEVs often preclude description of their function in the absence of physiological context, e.g., the stoichiometry of nEV-trafficked molecules needed to potentiate attributable biological effects is rarely considered. Instead, typical studies extrapolate function based on characterizations of the contents of nEVs enriched from cell culture media. It remains a great challenge to tease out the complex and likely intertwined autocrine, paracrine, and waste pathways of nEVs *in vivo*.

The compositional and temporal heterogeneity of nEVs enriched from *in vitro* but especially *in vivo* sources present significant challenges (Lacroix et al., 2012; Erdbrügger and Lannigan, 2016). Consequent irreproducibility of many nEV studies has led to a swath of attempts at standardization (Witwer et al., 2013; Coumans et al., 2017; Konoshenko et al., 2018), optimized isolation methods (Aatonen et al., 2014; Lobb et al., 2015; Xu et al., 2016), and comparative analyses across isolation and characterization methods (Rood et al., 2010; Tauro et al., 2012; Andreu et al., 2016; Rezeli et al., 2016; Ding et al., 2018). The International Society of Extracellular Vesicles (ISEV) regularly updates useful position papers establishing guidelines for the minimum experimental information to categorize nEVs and how to design effective control experiments for nEV functional analysis (Lötvall et al., 2014; Witwer et al., 2017; Théry et al., 2018). Especially relevant is the online knowledgebase EV-TRACK, developed to centralize data (Consortium et al., 2017). Notably, much of the work reviewed below is not present on EV-TRACK, nor meets the minimum guidelines suggested by experts, on-going issues that raise major concerns regarding stringency and reproducibility.

### Liquid Biopsy of nEVs

There is high diagnostic and prognostic potential for molecular profiling of aberrantly-expressed biomolecules comprising circulating nEVs, which are numerous and stable in peripheral blood and other biofluids (Revenfeld et al., 2014; Jakobsen et al., 2015; Lai et al., 2017). Diagnostics platforms for profiling circulating nEVs comprise three steps: (1) isolation/enrichment, (2) EV sub-fractionation via capture or other manipulation (e.g., immunoaffinity, microfluidic separation), and (3) analyte detection/fingerprinting. Isolation and enrichment techniques vary widely and have been extensively reviewed (Konoshenko et al., 2018), with differential ultracentrifugation (UC), density gradient UC, size-exclusion chromatography (SEC), and commercial PEG-based precipitation kits among the most commonly applied. It is clear isolation methods must be carefully considered and ideally varied within a single study (Théry et al., 2018). Pre-analytical surface immuno-capture using antibody (Ab) decorated surfaces are widely-used, feasible, and can be highly-multiplexed (Pugholm et al., 2015). For nEV detection, many studies quantify number or total average amount/composition with coarse, imprecise methods such as western blot (WB) or BCA assay. Nanoparticle tracking analysis (NTA) and flow cytometry use light scattering to generate particle counts, which have been correlated with cancer and disease progression (van der Vlist et al., 2012; Vestad et al., 2017), but require a large number of particles (>10^6^ per mL). Furthermore, detection based on light scattering is typically incapable of absolute sizing of nEVs smaller than ~70 nm, despite TEM evidence that a major fraction of EVs are smaller (van der Pol et al., 2018; Zhang H, et al., 2018). Fluorescence triggering, particularly for flow cytometry, has been applied to reduce the size limitation of light scattering approaches to ~40 nm (Arraud et al., 2015; Erdbrügger and Lannigan, 2016). Yet, fluorescence detection (including for direct imaging techniques like PALM/STORM) is susceptible to artifacts like blinking or bleaching, is limited in multiplexing capability due to broad overlapping fluorophore emission profiles, and ultimately depends on the effectiveness of fluorophore labeling, which is known to exhibit artifacts (Takov et al., 2017; de Rond et al., 2018).

More sensitive analytical biosensors are required for detecting small or rare events, critical for diagnosing early stage tumor formation or recurrence. Some promising candidates include optical resonators (Su, 2015), interferometric imaging (Daaboul et al., 2016), lens-free holographic microscopy (McLeod et al., 2015), and electrochemical sensors, often based on enzyme-linked immunosorbent assay (ELISA) or sandwich-ELISA (Jakobsen et al., 2015). While these methods have proved useful for downstream phenotyping of disease-specific nEV signatures, they are often limited by (1) lack of cost-effectiveness, (2) requirements for laborious preanalytical isolation and purification, (3) lack of high throughput detection/automation, (4) requirement for large sample volumes (i.e., low sensitivity), and/or (5) lack of multiplexibility (i.e., low specificity) (Pugholm et al., 2015). Many of the approaches reviewed below are focused on addressing these issues using nanoplasmonic platforms.

## Nanoplasmonic Techniques

**Figure 2:**
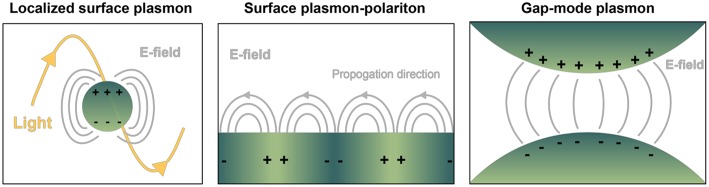
Depiction of common geometry-dependent plasmon modes for sensing biologicals. Localized surface plasmons (LSPs) originate when interrogating light interacts with nanoparticles or nanovoids of certain shape. Gray lines pictured represent electric fields. In contrast, on planar sensor surfaces incident light is capable of provoking pure evanescent modes by way of surface plasmon polaritons (SPPs), propagating waves that exponentially decay away from the interface of dielectric materials. LSPs exist over a finite frequency range, and they can be directly coupled with propagating light. SPPs occur over a wide frequency range, and they cannot be directly coupled with propagating light. Gap-mode plasmons typically comprise two metallic nano-features with a nanoscale gap in between. The gap region will exhibit a confined and intense light spot when the irradiating field is linearly polarized along the vector connecting the particles. Molecules confined in each of the resulting electric fields can experience a boost in signal detection compared to their native state, with great benefit to biosensing.

Nanoplasmonics encompasses the study and use of the unique light-matter interactions at the nanoscale exhibited by metallic structures, including metal nanoparticles (NPs) and metal substrates with nanoscale surface roughness (Jackman et al., 2017). These techniques are increasingly being applied for liquid biopsy of circulating biomolecules, notably for cancer diagnostics (Ferhan et al., 2018). From an electromagnetic point of view, metals can be considered a plasma, characterized by the free movement of conduction electrons throughout the bulk material. External electromagnetic fields (i.e., light) impinged on plasmonic materials can couple with the conduction electrons oscillating as waves along a metal surface, giving rise to a new entity with properties of both waves, known as *surface plasmon polaritons* (SPPs or just SPs). These are referred to as *localized surface plasmon resonances* (LSPRs) when the SP is confined to a nanoparticle surface with dimensions far below the wavelength of incident light. Particularly strong electromagnetic fields can be formed at the interface where two SPs meet, known as a *gap-mode plasmon* (Figure 2). With enormous benefit to imaging and sensing, SPs only occur at quantized frequencies, or modes, which can be tuned at will via precise control over size and shape of the material, in addition to its dielectric properties and those of the surrounding environment (Ringe et al., 2012). Static measurements of SPs (and their fluctuations in response to stimulus, analyte binding, etc.) have enabled the use of metallic substrates or NPs as versatile molecular detectors in many systems, including DNA hybridization, trace chemical sensing (Anker et al., 2008), and scanning near-field optical microscopy (Hermann and Gordon, 2018).

Biosensors based on SPs have many advantages, with potential for label-free quantitative analysis, a high degree of multiplexing, and ample potential for miniaturization (Lopez et al., 2016). These technologies may offer significant insight into nEV structure, function, and behavior, as (i) their size does not require ground-breaking sensitivity to observe single binding or sensing events (Zeng et al., 2019), (ii) most nanoplasmonic setups are realized as optical imaging platforms that can be easily integrated with fluorescence microscopy for increased multiplexing or direct imaging, (iii) the rapid timescale of plasmonic phenomena, combined with ongoing technical improvements allow for real-time tracking of nEV motion and interactions, and (iv) labeling-approaches where nanoplasmonic materials are bound to targeted EV subpopulations (e.g., via antigen binding) may have the advantage of subdiffraction imaging, effectively increasing spatial resolution (Hermann and Gordon, 2018). While the topic of plasmonic approaches to analyze EVs has already been reviewed (Im et al., 2015; Shpacovitch and Hergenröder, 2018), the field is rapidly changing, and this report is more focused on providing a detailed summary and critical feedback for each of the studies reviewed.

### Surface Plasmon Resonance Spectroscopy (SPRS)

Surface plasmon resonance (SPR) as an experimentally observed phenomenon dates back to the late 1960s, based on the understanding that polarized light is capable of exciting electrons residing at the interface of a dielectric and conductive metal surface (Kretschmann and Raether, 1968). Notably, rather than being an arbitrary event, SP coupling occurs only at well-defined physical circumstances whereby the wave vector of the incident light and the wave vector of the surface plasmons are matched, termed the “resonance condition.” The exact physical description of the resonance condition is derived from Maxwell's equations and can be readily calculated over a range of physical or optical conditions (Hayt and Buck, 2001; Maier, 2007). The key to understanding how one can exploit SP phenomena to measure analytical signals of biomolecules and their assemblies is to consider the refractive index (RI) of the volume where the SPs are travelling. Essentially, the particular resonance condition for a given plasmonic configuration is extremely sensitive to changes in RI within a nanometer-sized characteristic length scale from the material. Via general or target specific binding of biomolecules or their assemblies (e.g., nEVs), subtle alterations in RI give way to large observable changes in resonance condition, which is the essence of the sensitivity, or gain, for SPR/LSPR techniques. In recent years, there has been tremendous progress in the synthesis and use of plasmonic nanostructures that maximize the sensitivity toward biological reagents (Jans and Huo, 2012; Chinen et al., 2015; Liu et al., 2017; Zhang Y, et al., 2018).

#### Physical Background of SPR/LSPR

From an experimental viewpoint, SPR is used to detect events at the vicinity of the metal surface. In a typical SPR setup (Kretschmann-configuration) a prism is tightly connected via a refractive index matching material to a glass sensor chip coated with a thin (~50 nm) film of gold, though many other configurations exist, comprising diffraction gratings, optical fibers, and optical waveguides (Skivesen et al., 2007; Zhu et al., 2015; Michel et al., 2017). Interactions between an immobilized partner (ligand) and unknown sample (analyte) lead to changes of refractive indices and layer thicknesses within the reach of an *evanescent field* at the close proximity of the sensor surface. This field extends a small distance from the metal surface into the analyte solution with exponential decay over ~200–400 nm from the metal surface in conventional SPR platforms (Ekgasit et al., 2004). While interaction kinetics occurring very close to the sensor surface can be measured and quantified very accurately, it is critical that the binding region between the ligand and analyte resides within the region of highest evanescent field intensity.

**Figure 3:**
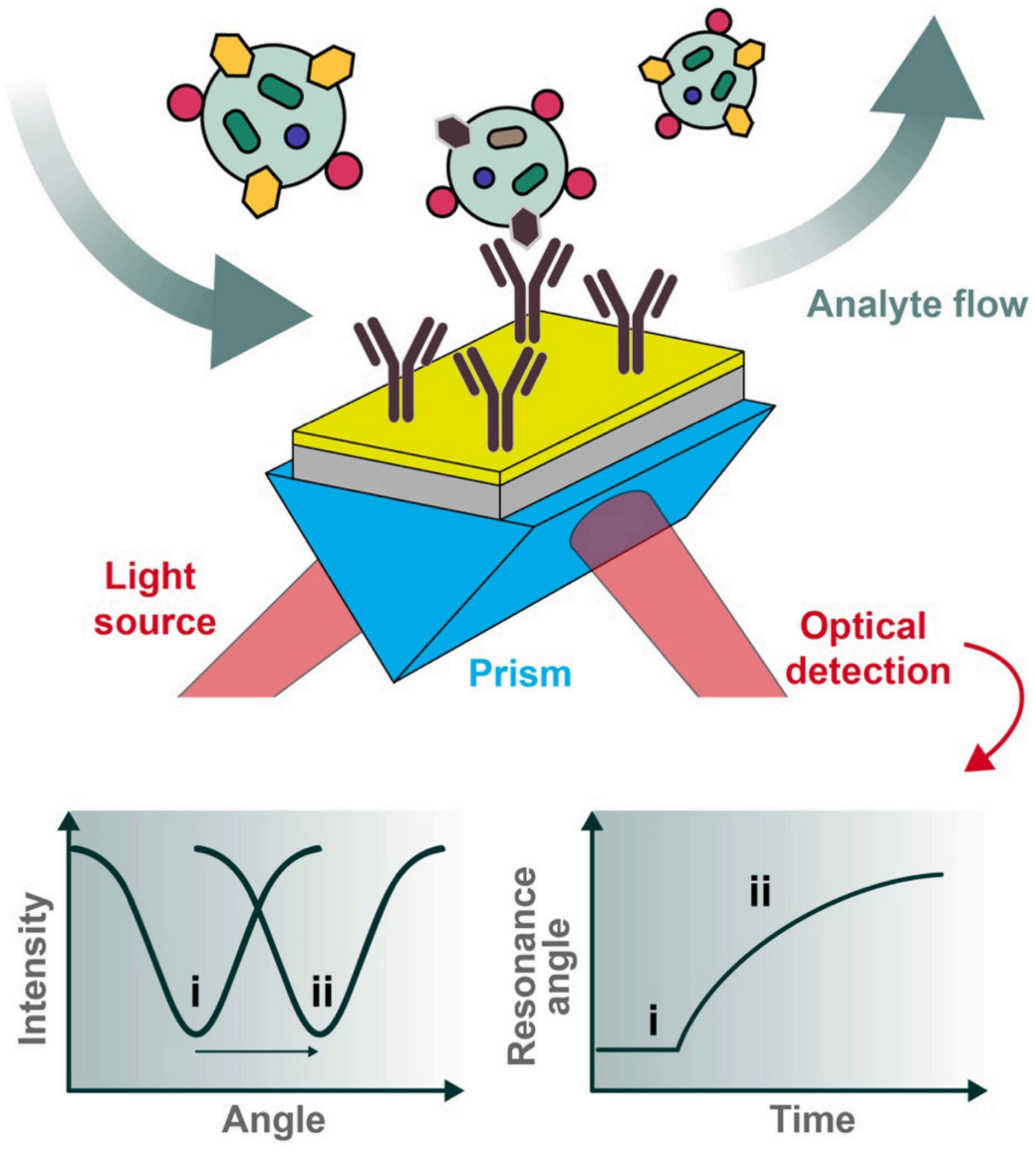
Typical set-up for an SPR biosensor. Polarized light is directed through a prism coupled to an interface between a metal (e.g., gold) and medium in contact with it. For biosensing applications, the sensor chip can be pre-functionalized with desired capturing ligands (e.g., antibodies) and the analytes of interest introduced under flow conditions. The reflected photons shift in angle of minimum intensity (resonance condition) upon changes in refractive index at the plasmonic surface (e.g., as a result of analyte binding), to be collected at the detection unit. Two types of graphs can be recorded, one being the reflected light intensity vs. the resonance angle peak shift (**left**) and the other representing the relative change of the resonance angle peak position vs. time (**right**). The two cases (i and ii pictured above) represent the before and after conditions of analyte binding, respectively.

In a *reflectance plot*, surface reflectance (*I*_*r*_) is measured as the incident light angle θ_0_ is varied. As the incident light angle reaches the surface plasmon resonance condition, the energy transfers from photons to surface plasmons, and the resulting resonance can be observed as a reflectance minimum (Figure 3). Another common visualization, known as a *sensorgram*, depicts the relative shift of the resonance angle as a function of time, which can produce interaction kinetics between a ligand and analyte(s) of interest using appropriate mathematical fitting.

**Figure 4:**
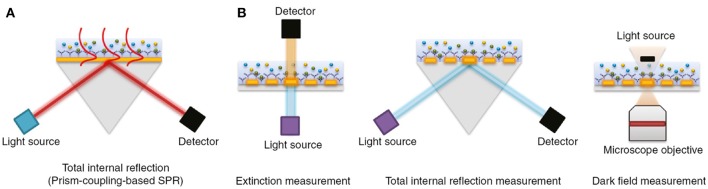
The most common biosensor configurations. **(A)** An SPR scheme based on total internal reflection (TIR) and the typical prism-coupling (Kretschmann configuration) which is needed for transverse magnetic (TM) modes of surface plasmon polariton excitation. **(B)** An LSPR extinction, TIR, and dark field measurement schemes for nanoarray surfaces, respectively. Figure reproduced as is from Lopez et al. (2016) under BY-NC-ND 3.0.

The physical principles of LSPR are fundamentally similar to SPR-based sensing. Instead, while SPR is typically carried out using planar metal substrates, LSPR relies heavily on the utilization of metal nanoparticles suspended in solution or micro- and nano-fabricated metallic structures such as gratings, nanopillars, and nanoarrays (Willets and Van Duyne, 2007; Potara et al., 2011; Ho et al., 2012; Ferhan and Kim, 2016). Figure 4 depicts common configurations for SPR compared to LSPR.

**Figure 5:**
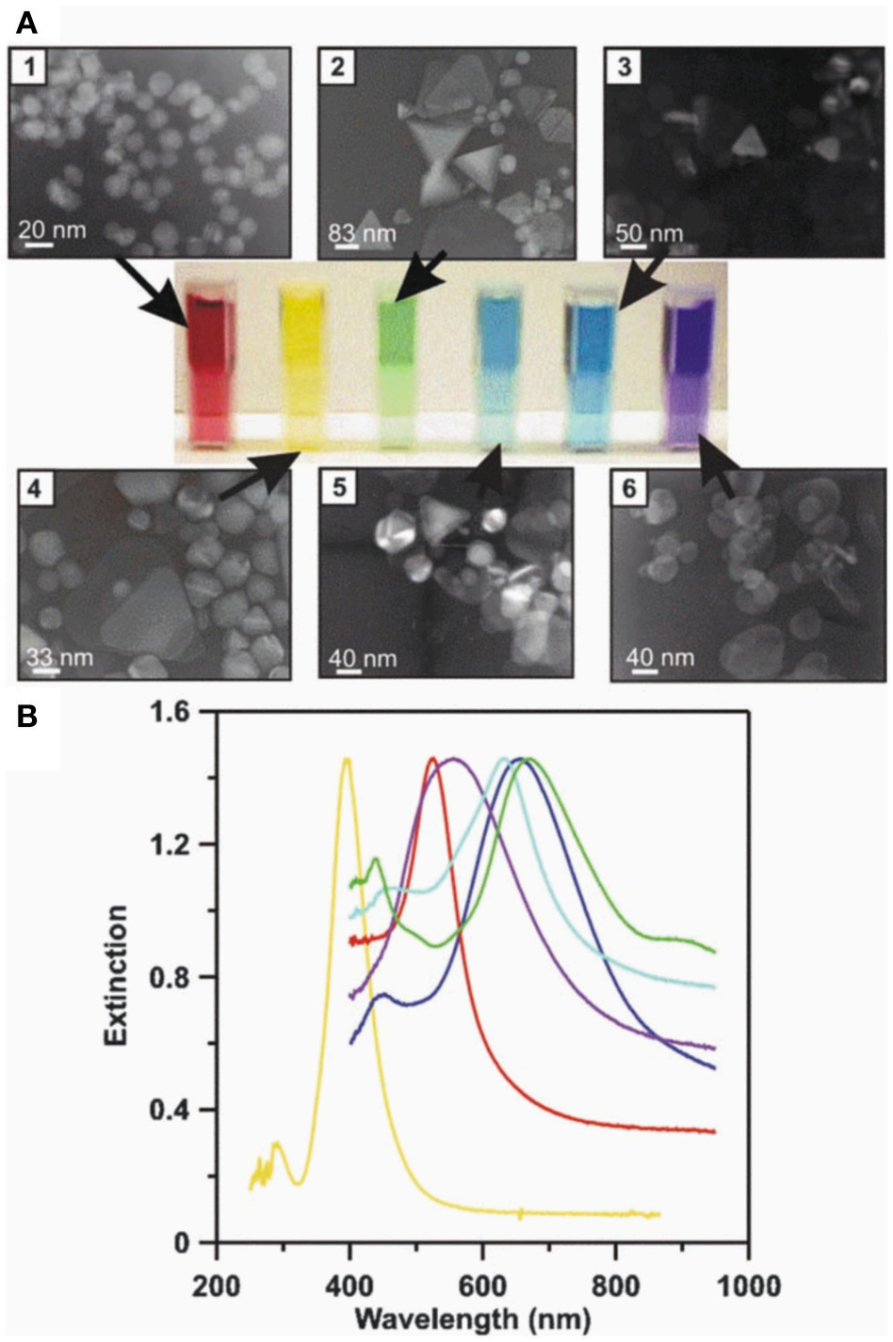
LSPR naked eye detection and extinction spectra for various Ag and Au NP solutions. A concrete representation of an LSPR measurement carried out for different sized and shaped metal NPs. **(A)** The transmission electron micrographs (1–6), corresponding metal NP solutions (from red to blue), and measured UV-Vis extinction spectra for their solutions. **(B)** The solutions' and spectrums' color schemes are paired. By modifying the physicochemical attributes of metal NPs, their respective LSPR conditions can be tuned to line up with desired technical settings (e.g., the selection of laser wavelengths) to achieve the best possible sensitivity and selectivity for a given assay. Reprinted by permission from Springer Nature (Haes and Van Duyne, 2004)^©^ 2004.

For LSPR, the photons of excitation light interact with metal NPs (or metallic structures with nanoscale features) to create a non-propagating free electron oscillation in the conduction band of the metal. As discussed in the context of SERS below, a remarkable electric field enhancement at the close proximity of the metal nanoparticle/structure can be observed. At the key plasmon resonance frequency, the absorption and scattering of light by the metallic nanostructures takes place (Willets and Van Duyne, 2007). A change to the local dielectric environment close to the nanostructure alters the polarizability, in turn shifting the plasmon resonance frequency and the optical extinction spectrum, an average of absorbed and scattered light collected by a detector (e.g., CCD or spectrophotometer) (Stewart et al., 2008). Particular attention is paid to the LSPR peak wavelength, where the highest light extinction is observed (Ruach-Nir et al., 2007). Plasmonic NPs can be highly tuned in size and shape to achieve desired resonance frequencies (Figure 5) (Haes and Van Duyne, 2004), offering an intriguing window for biosensing and even allowing naked eye detection (Haes et al., 2004; Chen et al., 2008). In LSPR, the enhanced electric field distribution is located at the vicinity of the metal nanostructure, therefore the optimal sensing distance is ~10–30 nm.

Gold is the most preferred material of choice for LSPR for several reasons: (i) it is relatively inert, (ii) thiol chemistry enables straightforward functionalization, and (iii) its plasmon frequency allows for detection using inexpensive UV-Vis spectrophotometers (sometimes even an unaided eye) (Sepúlveda et al., 2009; Hill, 2015; Unser et al., 2015). Other nanoplasmonic materials, like titanium nitride (TiN), have also been applied for nEV analysis (Qiu et al., 2019).

**Figure 6:**
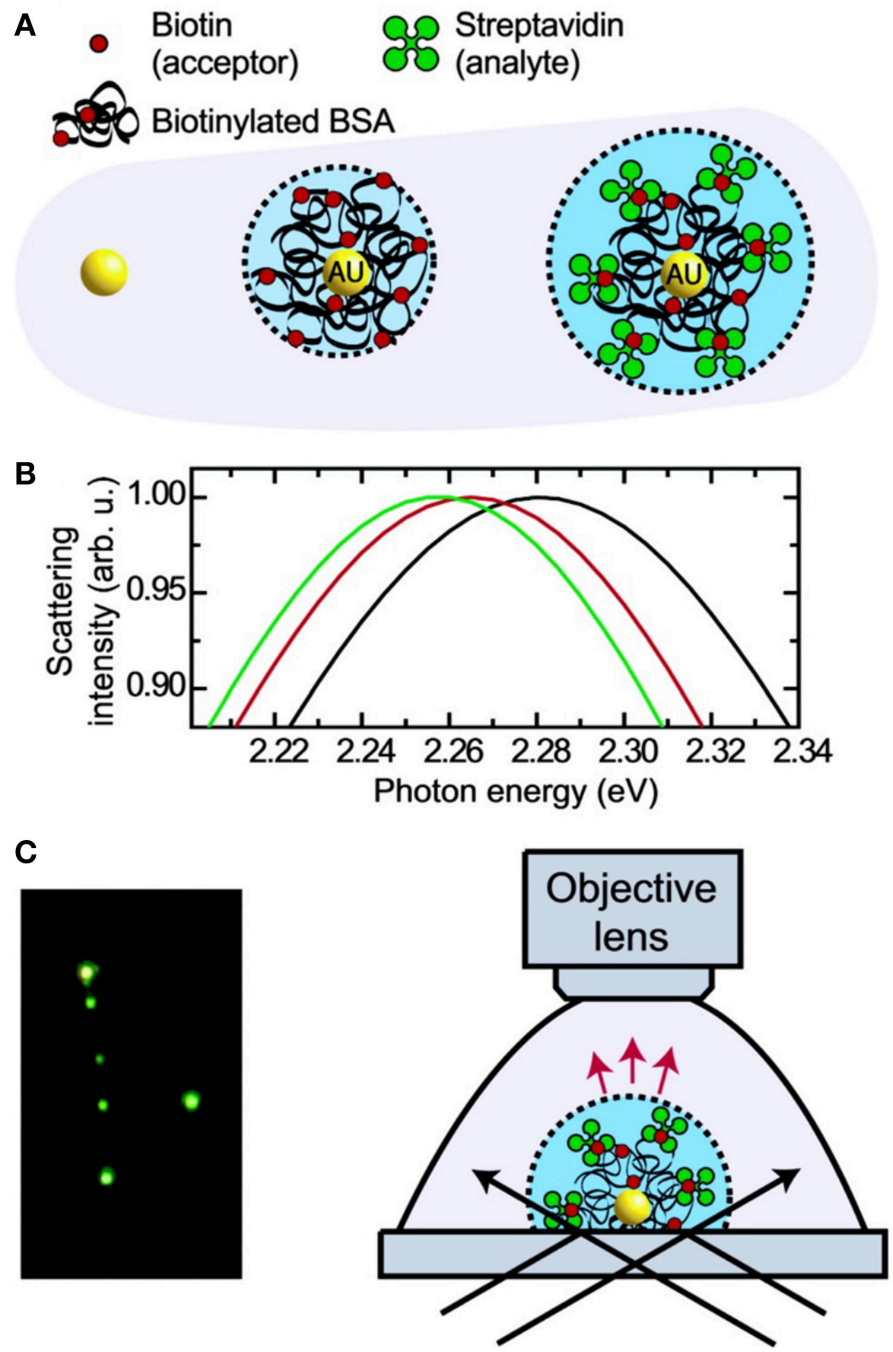
Principle and schematic representation of a biosensor based on light scattering from a single AuNP. **(A)** Single AuNPs are functionalized with biotinylated BSA protein which subsequently binds streptavidin. **(B)** Mie theory calculations for the three different environments shown in **(A)**. **(C)** Left: true color photograph of a sample of functionalized AuNPs in dark- field illumination. Right: experimental setup facilitating dark-field microscopy of single AuNPs immersed in liquids. Reprinted with permission from Raschke et al. (2003). Copyright (2003) American Chemical Society.

Typically, a LSPR biosensing experiment is carried out by measuring the background, for example blank buffer solution, and consequently the sample of interest. Prior to detection, sensing nanostructures are typically functionalized with various ligands (for instance antibodies, nucleic acid strands, receptors) to capture the analyte of interest. The non-functionalized surfaces and sites in the nanostructures are blocked by inactive components to avoid non-specific adsorption of co-analytes. Given that the target analyte (e.g., nEVs) typically has higher RI than the blank background, the local dielectric environment near the plasmonic nanostructure experiences a change, which can be seen as the LSPR peak shift toward higher wavelength (Willets and Van Duyne, 2007; Unser et al., 2015). This “red shift” corresponds to the concentration of nanostructure-bound target molecule (or particle), hence enabling quantitative measurements (Figure 6) (Raschke et al., 2003). However, low target concentrations remain a challenge, since RI-based sensing produces relatively small peak shifts, typically <10 nm (Guo et al., 2010). A secondary probe may be used to amplify such signals, for instance a gold nanoparticle (AuNP) conjugate (Hall et al., 2011). As a result, the electromagnetic field becomes more enhanced and the LSPR peak undergoes a significant broadening along with an immense shift, of ~50–100 nm, thus a clear shift e.g., from red to blue color, can be perceived (Elghanian et al., 1997; Chen et al., 2010; Xia et al., 2010). LSPR detection has also been combined with ELISA for detection of tumor and viral markers at ultralow concentrations reaching the attomolar level (de la Rica and Stevens, 2012).

#### Applications of SPR/LSPR to nEVs

##### SPR analytical method

Among the first reports of SPR to determine nEV concentration in solution was carried out via capture of CD63(+)-nEVs with surface-immobilized antibody (Rupert et al., 2014). The fundamental finding was to relate the surface-bound mass increase (via the SPR response) over time to the nEV concentration in solution. Using nEVs isolated from human mast cell (HMC-1.2) culture supernatant by UC, the accuracy of concentration quantification was found to be influenced by (i) the broad nEV size distribution and (ii) the structural changes nEVs may undergo upon the binding event to the antibody-functionalized surface. Control and calibration efforts with proteins and synthetic lipids were made to address these issues, and comparison to NTA and bicinchonic acid (BCA)-based quantification methods was made. Ultimately, when the size dispersity was considered, the SPR-based concentration determination yielded ~2-fold larger concentrations than the BCA assay indicated. In this regard, the authors assumed that the nEV preparation was free of contaminating protein and that nearly every vesicle expressed CD63. The authors concluded that the measured nEV mass concentration resides within a reasonable range between 2 and 16 μg/mL, derived by making rather radical assumptions regarding the composition, buoyant density, and membrane permeability of the nEVs. One of the foremost biochemical simplifications was that CD63 expression alone sufficiently represents the overall nEV capturing efficiency of the designed biosensor. The authors followed up with another study employing an SPR instrument equipped with two excitation wavelengths (670 and 785 nm) and a scanning angle feature (Rupert et al., 2016). To estimate the sizes of surface-bound liposome controls and the bulk concentration of CD63(+) nEVs, a rigorous mathematical SPR formalism was introduced for films, spherical nanoparticles, and spherical or deformed shell structures binding to the sensor surface. This, in turn, made it possible to achieve better estimates of the contribution of nEV binding-induced deformation on the bulk concentration determination. The key finding was that a dual-wavelength SPR system allowed for determining thicknesses of bio-films comprised of nanoscale particles. The sources of error of the nEV bulk concentration overestimations made in the previous study were successfully traced to mainly stem from the binding-induced deformation. However, it is to be noted that the introduced methodology probed only the subpopulation of CD63(+) nEVs, corresponding to ~5–10% of the entire EV sample. Another shortcoming of this pioneering work was that a direct comparison between the NTA (particles/mL) and total protein content (μg/mL) is clumsy. Firstly, converting the number of particles per volume (NTA) to mass per volume is affected by a plethora of uncertainties and second, NTA and BCA measurement are each sensitive to the impurities present, e.g., soluble protein aggregates, yet to greatly varying degrees.

Recently, a robust, real time, label-free SPR biomarker detection platform for ICAM-1(+) nEVs was developed for predicting the existence and stage of coronary heart disease (CHD) (Hosseinkhani et al., 2017). UC, SEC, and commercial precipitation kits (ExoQuick-TC™) were tested for EV isolation from cell culture supernatants. The optimized SPR detection platform employed surface-immobilized antibodies: anti-ICAM-1, anti-CD63, and anti-IgG1, which could distinguish ICAM-1(+) nEVs released under simulated inflammatory stress. With the motivation of avoiding tedious sample purification and labeling steps, as well as accomplishing a robust and sensitive detection of nEV subpopulations, the described SPR methodology was successfully employed using low sample volume consumption and without the need for (external or internal) calibration. Unfortunately, neither the limit of detection (LOD) nor limit of quantification (LOQ) were assessed. Also, a concern can be raised in terms of pinpointing ICAM-1 expression as an adequate biomarker for CVD prognosis and diagnosis. These slight defects, however, do not take away from the appealing findings of using SPR for the purposes of EV-based biomarker detection.

Another study focusing on both cell line-derived and human blood-isolated nEV detection by SPR was carried out (Grasso et al., 2015). There the authors employed three divergent breast cancer cell lines, MCF-7, BT-474, and MDA-MB-231, to generate nEVs, isolated by a combination of low-speed centrifugation, ultrafiltration (UF), and further purification by SEC. Plasma nEVs followed the same protocol excluding UF steps. A multiplexed SPR assay approach with two typical exosomal (CD9 and CD63) and four cancer-specific (CD24, CD44, EpCAM, and HER2) biomarkers was demonstrated, with a prerequisite of sample volume in the range of 5–20 μL. In order to prevent non-specific binding, a self-assembled monolayer of carboxylated polyethylene glycol (PEG) polymer was first added to the gold surface, followed by neutravidin treatment and the biotinylated antibodies. Large variations in biomarker expression was observed across nEVs isolated from the three breast cancer lines, notably with modest CD9 and CD63 expression, abundant CD24 and CD44 expression, practically negligible EpCAM, and moderate HER2 expression levels. Despite commendable insights and findings, the research had a few caveats that experts in the EV field may want to take into consideration to improve upon this approach in future studies. Namely, (i) adequate controls e. g., in the form of non-cancerous nEVs, were lacking, (ii) rigorous and clear statistical treatment of the obtained results was insufficient, (iii) description of the SPR signal processing mathematics was ambiguous, (iv) only qualitative color-coded panel(s) of the main results were shown, (v) data were inconclusive with respect to the plasma-originating nEV profiling by SPR, (vi) little attention was paid to assay reproducibility, and (vii) LOD and LOQ were absent.

**Figure 7:**
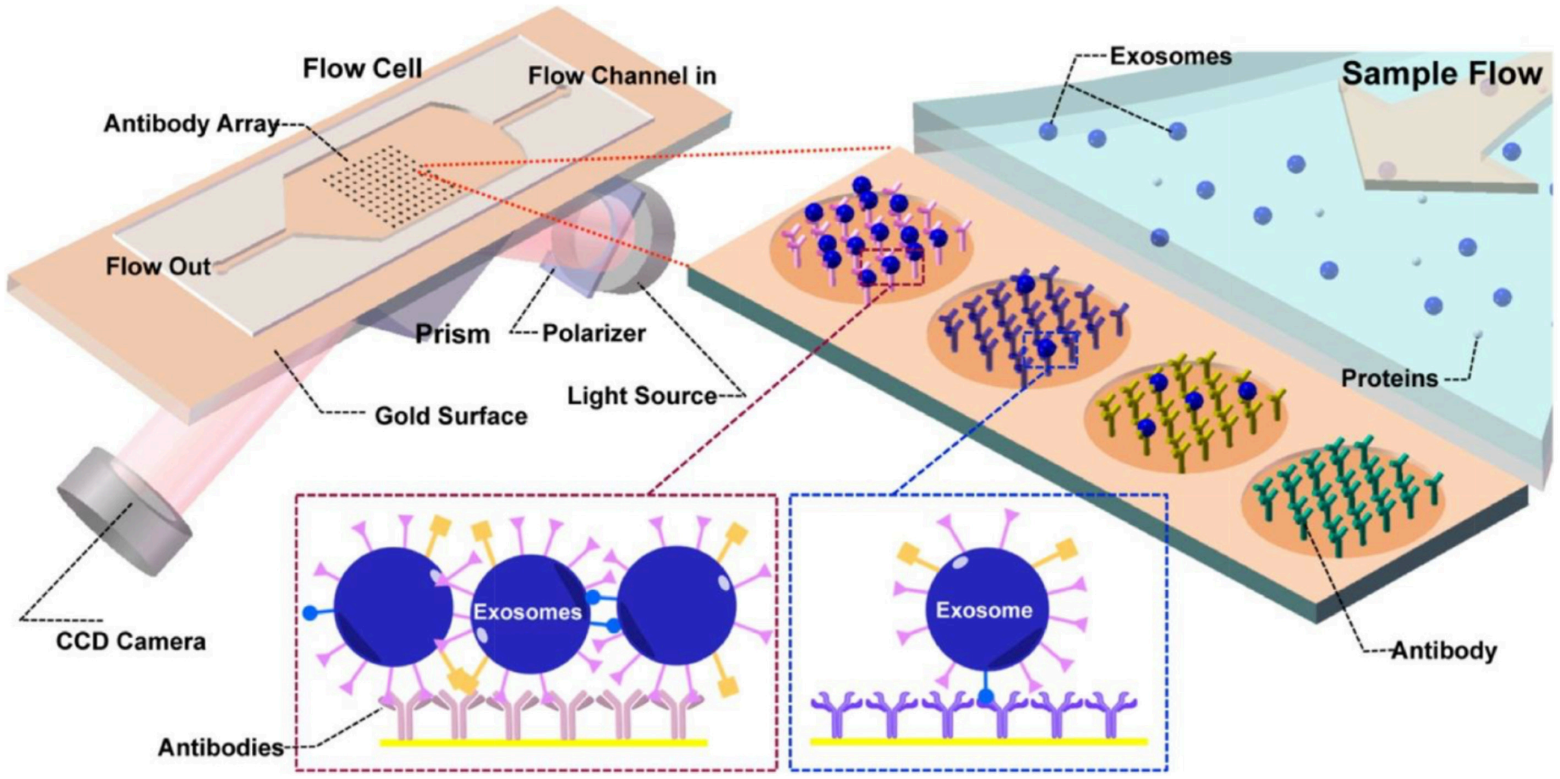
Principles of the SPR imaging (SPRi) system. The core of the flow cell is a functionalized Au biosensor comprising printed antibody regions whereby the capturing and detection of nEVs takes place. Prism coupling is used to excite the SPR, and the reflected light is collected by a CCD detector. Upon nEV binding, the local refractive index at the vicinity of the sensor surface increases. This detunes the resonant coupling of light to surface plasmon polaritons, and at the resonance condition, the resonance angle peak minimum is observed in the intensity of the reflected light. Consequently, the peak shift from before and after the affinity binding can be quantified to determine the interaction kinetics and surface-bound mass. Reprinted with permission reproduced from Zhu et al. (2014).

Taking advantage of the previously mentioned benefits of SPR, and expanding on the concept, an SPR imaging (SPRi) platform using multiplexed antibody microarrays was introduced (Zhu et al., 2014). The intention was to quantify EVs in tumor cell culture medium directly. Four different cell lines were analyzed: MHCC97H (human hepatocellular carcinoma, highly metastatic), MHCC97H (human hepatocellular carcinoma, meagerly metastatic), B16-F1 (melanoma, highly metastatic), and B16-F10 (melanoma, meagerly metastatic). EVs were either isolated by UC, or were measured in the supernatant directly. The SPR system comprised a light source irradiating the gold sensor surface through a coupling prism at a fixed angle position and a CCD camera for detecting the reflection and imaging the surface (Figure 7). Since the pixels on a CCD detector array are sampled concurrently, the reflected light intensity at each pixel can be easily monitored (Campbell and Kim, 2007). Therefore, the amount of surface-bound analyte at each spatial position can be accurately studied in a high-throughput manner. In essence, Zhu et al. harnessed these capabilities by printing microarrays of antibodies specific to transmembrane proteins of nEVs. The total palette of antibodies included ones against CD9, CD63, CD41b, CD81, CD82, E-cadherin, and EpCAM. A few anti-intracellular part antibodies were chosen to demonstrate the biogenesis pathway of nEVs: CD9 N-term, CD81 C-term, CD82 C-term, and E-cadherin C-term. Control antibodies used were against IgG, MET, in addition to HRP-conjugated secondary antibodies. The nEVs isolated from MHCC97H cell culture supernatant bound to all the expected antibodies, while negligible signal stemmed from negative control anti-IgG. Anti-CD9 and anti-CD41b demonstrated especially high binding capability, indicating the expression of corresponding membrane proteins on the investigated nEVs (and verified by WB). Measurements directly on unprocessed culture supernatant provided similar binding results with consistently high signals, suggesting that it could be directly used for nEV binding and transmembrane structure identification studies. Lastly, the changes in amounts of nEVs derived from MHCC97H cell culture supernatant were investigated. Secretion of nEVs was modified using siRNA-Rab27a transfection (hypothetically decreasing exosome-type secretion) and monensin treatment (hypothetically increasing secretion). When monitored by the SPRi microarray sensor chips, the binding signals to anti-CD9 and anti-CD41b were significantly higher in the non-siRNA transfected and monensin-treated groups, confirming the hypotheses.

With respect to the immense experimental and standardization efforts made in the EV field to resolve the most efficient methods for isolation and to assess the purity, the work by Zhu et al. represents a decidedly large leap forward, having deduced that the SPRi signals indeed originate from EV binding rather than free protein or membrane debris in the cell culture supernatant (Zhu et al., 2014). Although well-justified, a concern can still be raised, whether the high signals stem from non-exosomal debris in the supernatant. Several validation experiments would have been needed to strengthen the made observations. For instance, a useful control would have been EV purification by the density gradient method and comparison of signals between the “debris” fractions and “EV” fractions. Moreover, the EV characterization in general was short of size distribution data (NTA or DLS) or high quality TEM images.

Recent attempts have begun to appreciate the complexity of more clinically relevant nEV preps using label-free plasmonic approaches. A custom-made SPR instrument was used to detect nEV subpopulations amongst a heterogeneous sample isolated from patient serum (Sina et al., 2016). A sandwich approach on a gold surface was utilized; either biotinylated anti-CD9 or anti-CD63 was immobilized on streptavidin-coated surface to capture vesicles. Anti-HER2 was utilized as a detection antibody to identify breast cancer nEVs were isolated from cell culture medium using the commercial precipitation Total Exosome Isolation Reagent (TEIR). In the assay development phase, 10% HER2(+) nEVs could be detected when predetermined portions of HER2(+) BT474 and HER2(–) MDA-MB-231 cell-derived nEVs were used. A calibration curve based on the theoretical minimum and maximum sensitivities was calculated via titration of heterogeneous mixtures of the two aforementioned nEV types. The LOD was defined at 2,070 nEVs/μL. The authors further isolated nEVs from six HER2(+), two HER2(–) breast cancer patient serum samples, and two serum samples from healthy individuals. Bulk nEV capture onto the biosensor surface was independent of the capturing agent used (either anti-CD9 or anti-CD63) and the signal levels in the capturing phase were rather similar. Upon detection using anti-HER2, 14–35% of the bulk nEV population consisted of HER2(+) nEVs for the breast cancer patients. While the study did not have any obvious flaws, it would have benefited from slightly broader perspective, e.g., nEVs from other cancerous and non-cancerous cell lines or larger cohort of patients could be tested—which the authors also suggested themselves.

An intriguing concept combining LSPR and SPR was recently introduced (Di Noto et al., 2016). Monoclonal gammopathy of undetermined significance (MGUS) has been observed as a significant intermediate step in all cases of multiple myeloma (MM) and the role of nEVs in this step, as well as in the development of MM in general, has drawn interest. nEVs from 5 MGUS, 10 MM, and 10 healthy individuals were isolated by UC and further purified by sucrose gradient UC. The authors had previously perceived that heparan sulfate proteoglycans (HSPGs) on the cell surface are mediators in the nEV uptake by the target cells (Di Noto et al., 2014), where MM-derived nEVs were internalized more than nEVs isolated from MGUS and healthy individuals. Therefore, in order to differentiate these three nEV groups and to quantify their corresponding binding efficiencies, the researchers developed an SPR biosensor functionalized with heparin (a structural analog of heparan sulfates). As equivalent concentrations of nEVs were passed over the heparin-functionalized SPR biosensor, the binding of MM-isolated nEVs was consistently higher than in the two other groups. Thus, the SPR platform was shown to be a robust and label-free method to tease out differences between nEVs. Another incremental advancement to SPR was recently introduced using magnetic NP-enhanced nEV detection for grating-coupled SPR (Reiner et al., 2018). The system allowed for parallel SPR and plasmonically-enhanced fluorescence monitoring of MSC-derived nEVs, representing a novel usage of label-enhanced SPR and fluorescence in combination.

In conclusion, the innate features of SPR offer a range of opportunities for nanoscale detection and characterization of nEVs—typically by label-free manner. High tunability of Au-based sensors provide room for creativity in method development. The current setbacks that hamper bridging the gap between research and clinical applications mostly relate to the perceived complexity of SPR techniques, and rather bulky instrumentation. In terms of nEVs in particular, non-exosomal debris (lipoproteins, aggregated proteins, etc.) in samples exacerbate reaching adequate specificity and sensitivity as all these components may bind to biosensor surface and change the local RI at its vicinity. Advancements in optics (lasers, optical components, miniaturization capabilities) could be harnessed to address the instrument-related shortcomings. On the other hand, rapidly developing understanding of chemical modification of plasmonically-active materials may provide new insights for high-precision biosensor surface preparation (Jans and Huo, 2012). Another useful angle for the future of SPR work would be the adoption of an EV-mimicking standard for use as a control to model binding.

##### LSPR-based sensing

Generally, LSPR platforms consist of detecting analytes located near (i) metallic nanostructure arrays on a solid support generated using micro- and nano-fabrication lithographic techniques or (ii) nanoparticles, freely suspended in solution or deposited onto a solid support.

**Figure 8:**
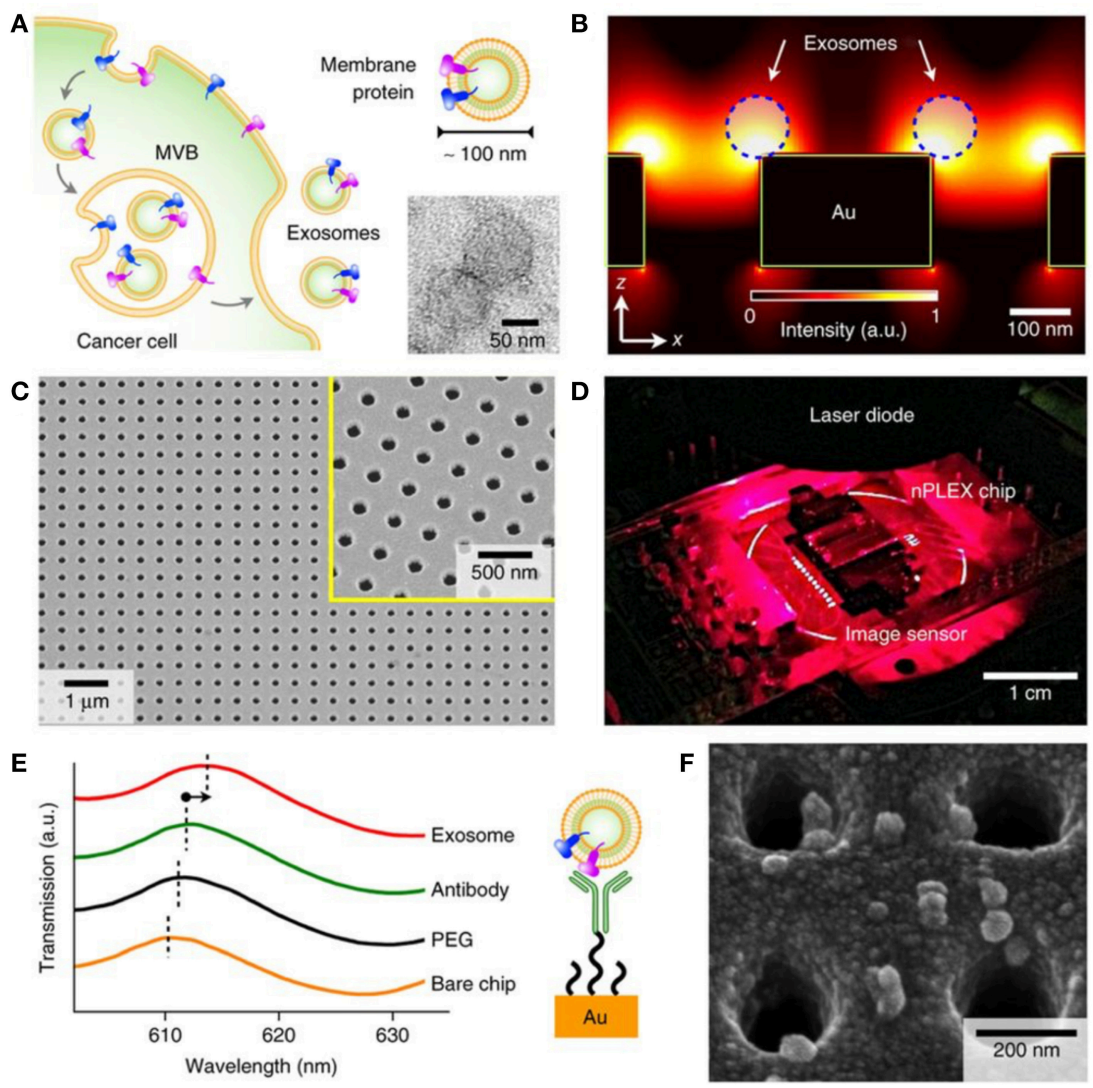
Label-free detection of nEVs using the nPLEX biosensor. **(A)** The well-defined biogenesis route of exosomes from cancer cells via multivesicular body (MVB) formation, consequent fusion with the cell membrane, and excretion of exosomes. The upper inset displays membrane proteins on the shell of the exosome; these proteins act as common recognition sites in biosensing applications. The lower inset shows a transmission electron micrograph of exosomes isolated from human ovarian cancer cell (CaOV3) culture. **(B)** Near a periodic nanohole surface the electromagnetic fields are clearly enhanced and accumulated as shown by finite-difference time-domain (FDTD) simulation. The enhanced field spreads over the nanostructure from one hole to another, which enables high sensitivity for the nPLEX assay. **(C)** The nPLEX sensor imaged by a scanning electron microscope (SEM). The nanoholes of diameter 200 nm are distributed evenly over the surface with periodicity of 450 nm (the inset demonstrates a closer view of the surface). **(D)** The nPLEX system is equipped with a metal-oxide-semiconductor (CMOS) detection unit for acquiring transmitted light intensity from the sensor chip. **(E)** The binding of nEVs to a periodic nanohole structure induces a change in the local refractive index and thus a spectral shift in transmission spectral peak. Wavelength shifts, or intensity changes at fixed wavelength, are monitored for nEV quantification. **(F)** An SEM image demonstrating surface-adsorbed exosomes on the nPLEX sensor. Reproduced by permission from Springer Nature (Im et al., 2014).

*Nanohole and nanopillar arrays*. The nano-plasmonic exosome (nPLEX) is an LSPR-based assay utilizing optical transmission through an array of periodic nanoholes patterned to a 200 nm thick gold film on a glass substrate (Figure 8) (Im et al., 2014). Sensitive high-throughput nEV analysis was accomplished by using nanohole dimensions approximating average nEV size. Detection was based on either spectral shifts or intensity changes that were induced by nEV binding to antibody-functionalized nanoholes. To control non-specific binding, nPLEX devices were pre-coated with a mixture of short- and long-chained PEG polymers. First, nPLEX assay protocol was established through examination and quantification of nEVs secreted by *in vitro* CaOV3 OvCa cells. A high-affinity binding constant of ~36 pM and LOD of ~3,000 exosomes (670 aM) was observed when the nanoholes were functionalized with anti-CD63, and subsequent capturing of CaOV3-derived nEVs was performed. In comparison to WB and ELISA, the nPLEX protein quantification performed better (sensitivities 10^4^ and 10^2^-fold higher, respectively). Notably, secondary labeling significantly amplified signaling of the nPLEX platform: spherical Au nanoparticles showed a 20% increase and larger, star-shaped Au NPs showed 300% amplification. Another OvCa cell line, OV90, was used in parallel with CaOV3 to compare the capabilities and correlation of nPLEX against ELISA (EpCAM, CD24, CA125, MUC18, EGFR, and HER2 were used as protein markers). Correlation of R^2^ > 98% was found. Concurrently, the captured nEVs were eluted from the nanostructures, and a quantitative real-time PCR (qRT)-PCR was implemented to investigate the mRNA contents of the nEVs. Next, the researchers did a molecular screening of nEVs encompassing various non-cancerous and cancerous cell lines. Subsequent observations were applied to a cohort of OvCa (*n* = 20) and cirrhosis (non-cancerous, *n* = 10) patients to determine the nPLEX performance. nEVs were enriched only using 0.2 μm membrane filtration. The amounts of EpCAM and CD24 were significantly higher in the OvCa group. Lastly, a small cohort (*n* = 8) of OvCa patients undergoing chemotherapy were profiled to evaluate patients' response to treatment. Levels of EpCAM and CD24 showed a decreasing trend in the corresponding patients. In future, the use of larger patient cohorts and additional disease models will strengthen the power and applicability of the platform.

In a similar study, nanohole-based surface plasmon resonance platform named intravesicular nanoplasmonic system (iNPS) was developed to screen both surface as well as intravesicular proteins trafficked by nEVs released from ovarian cells (Park et al., 2017b). The engineered substrates provided a sensitivity capable to detect nEVs in as little as 0.5 μL sample per marker in a high-throughput manner using a 10 × 10 array. Another nanohole array assay was used for detection of pancreatic ductal adenocarcinoma (PDAC) (Yang et al., 2017). The sensing scheme was based on measuring the resonant light transmission—more specifically quantifying the spectral shifts—upon binding of tumor-derived nEVs to a periodic array of antibody-functionalized nanoholes. A 100 nm thick Au layer with nanoholes of 200 nm in diameter and periodicity of 500 nm was constructed. Platform calibration and validation was via a PDAC nEV panel on a training cohort of 32 patients and healthy controls. As a result, a five-marker PDAC^EV^ signature comprising EGFR, EpCAM, MUC1, GPC1, and WNT2 components was established (accuracy reaching 100%) and further validated with a cohort of 43 human samples, with an accuracy of 84%, sensitivity of 86%, and specificity of 81%. A correlation study was performed between the PDAC^EV^ signature and gold standard markers (CA19-19 and CEA), finding none. Sixty-one percent of PDAC patients showed elevated CA19-19 levels and 17% of PDAC patients showed elevated CEA levels, while PDAC^EV^ values revealed 89% of PDAC patients. High specificity and sensitivity, scalability, throughput, automated operation, molecular printing capabilities, cost effectiveness, and low sample consumption were the advantages of the developed method. Meticulous statistical analyses complemented the different phases of the study and underlined the high translation potential of the platform.

Two-dimensional (2D), quasi-three-dimensional (quasi-3D), and 3D plasmonic photonic crystal (3D PPC) nanostructures have been developed and evaluated for nEV detection (Zhu et al., 2018). For 2D gold nanoholes, SPs were found to localize at the edges of the nanoholes. Asymmetrical Au nanoholes showed a higher electromagnetic field intensity and therefore higher sensitivity. Quasi-3D Au nanoholes consisting of Au nanoholes on top and Au nanodots at the bottom produced an even greater EM field intensity due to the hybrid coupling of LSPR and Fabry-Perot modes. The 3D PPC nanostructure was created by adding an additional array of Au nanosquares to the top of the quasi-3D nanoholes. This served to further strengthen the EM field and increase sensitivity. Target nEVs were enriched by UC from fibroblast L cell lines for detection using the 3D PPC platform, first coated with anti-EpCAM antibody for nEV capture. Detection range was measured to be between 10^4^ and 10^11^ particles/mL. While the study had its impressive merits from the nanomaterials development and physics point of views, there are some notable oversights. First, nEV concentration was measured only by NTA, and subsequently used as an absolute value for sensitivity assessments. The authors did not report complementary nEV quantifications (e.g., WB or TEM), nor assess purity of the preps. A concern can be raised, whether the speculated *intravesicular filaments* pictured in the final image of the study are due to non-nEV impurities, thus resulting in the larger plasmon sensing area, which was considered to contribute to the higher sensitivity of the 3D PCC nanostructures. A second suggestion would be to measure several additional sources/types of nEVs for validation.

**Figure 9:**
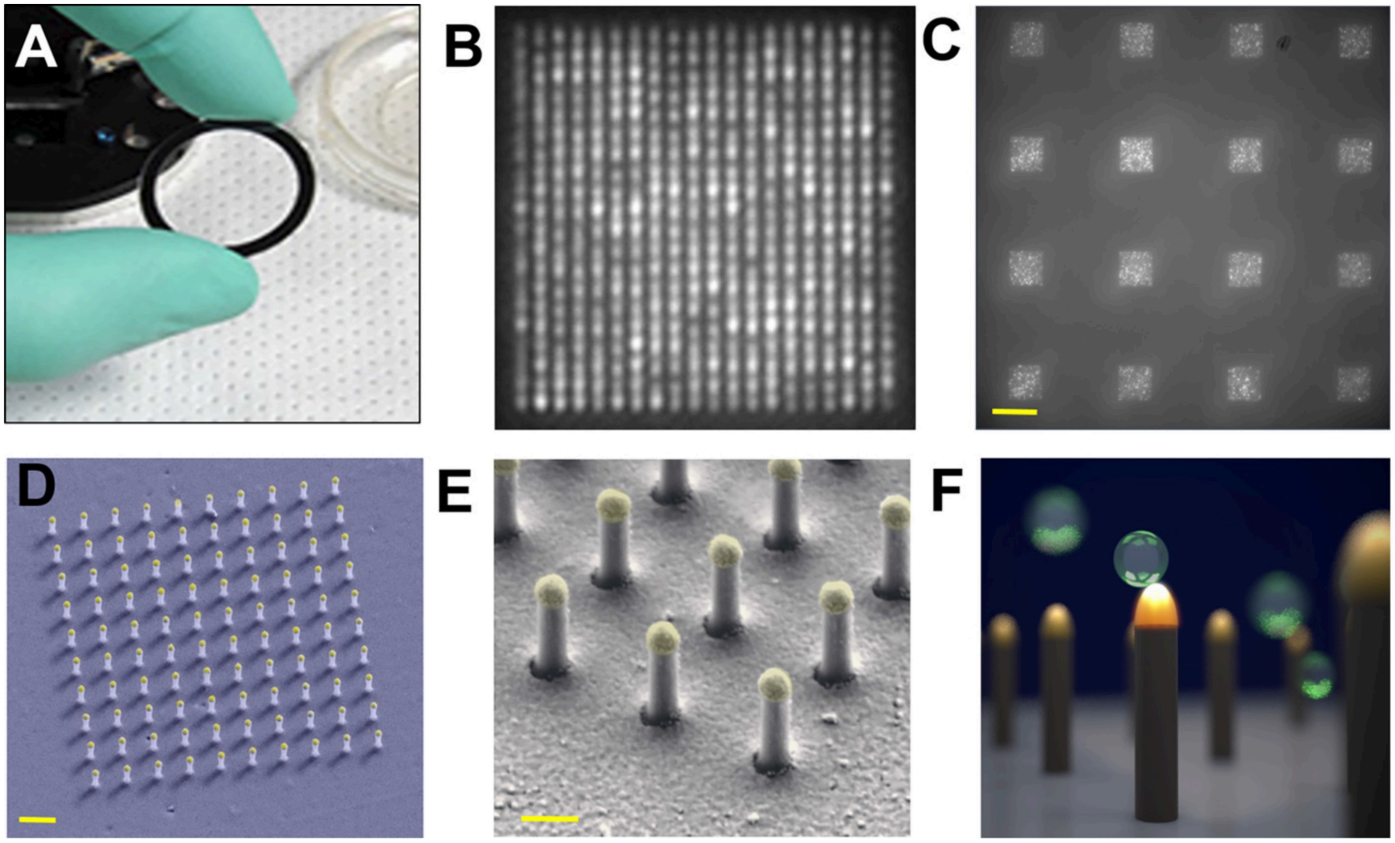
Nanoplasmonic pillars. **(A)** 25.4 mm diameter LSPRi sensor chip. **(B)** LSPRi image of a 20 × 20 array, pitch size of 600 nm, scale bar: 1 μm. **(C)** LSPRi image of sixteen arrays in the field-of-view taken using 100X/1.4 NA objective, each consisting of 400 plasmonic nanopillars in a 20 × 20 square lattice and 500 nm pitch, scale bar: 10 μm. **(D)** False colored SEM image of a 10 × 10 nanopillar array, scale bar: 1 μm. **(E)** High-magnification false colored SEM image showing detailed view of individual nanopillars, scale bar: 200 nm. **(F)** Diagram illustrating size matching of individual nanopillars diameter (*d* = 90 nm) to that of nEVs (~50 nm < *d* < 200 nm), allowing digitized vesicle detection while also elevating the sensor to minimize background contributions from the substrate. Figure reproduced under Creative Commons License 1.0 from Raghu et al. (2018).

Single nEV detection was pursued by a LSPR imaging (LSPRi) system (Raghu et al., 2018). The sensing elements have Au caps topping a matrix of quartz nanopillars, the latter being relatively inert to non-specific binding. The individual nanopillars (total height 497 nm with diameter ~90 nm) were assembled together into an evenly spaced 10 × 10 or 20 × 20 array chip for high multiplexity (Figure 9). A CMOS camera was capable of imaging 6,400 nanopillars (16 arrays with 400 nanopillars each) with a resolution of 0.36 μm^2^ per nanopillar, while spectra were simultaneously acquired with a CCD-based spectrophotometer. nEVs were isolated from MCF7 breast adenocarcinoma cells by commercial precipitation kits. Roughly 1 × 10^5^ nEVs/mL (by NTA) were introduced to the LSPRi sensors using a microfluidic system. The authors state that single-nEV binding events are resolvable from free protein as a result of the subsequent discrete step function response in comparison with a smoother integrated response that smaller analytes would produce. For this work, it is noted that precipitation kits may induce artifacts to the observed signals in the form of aggregated nEVs or larger non-EV contaminants due to the isolation reagent.

**Figure 10:**
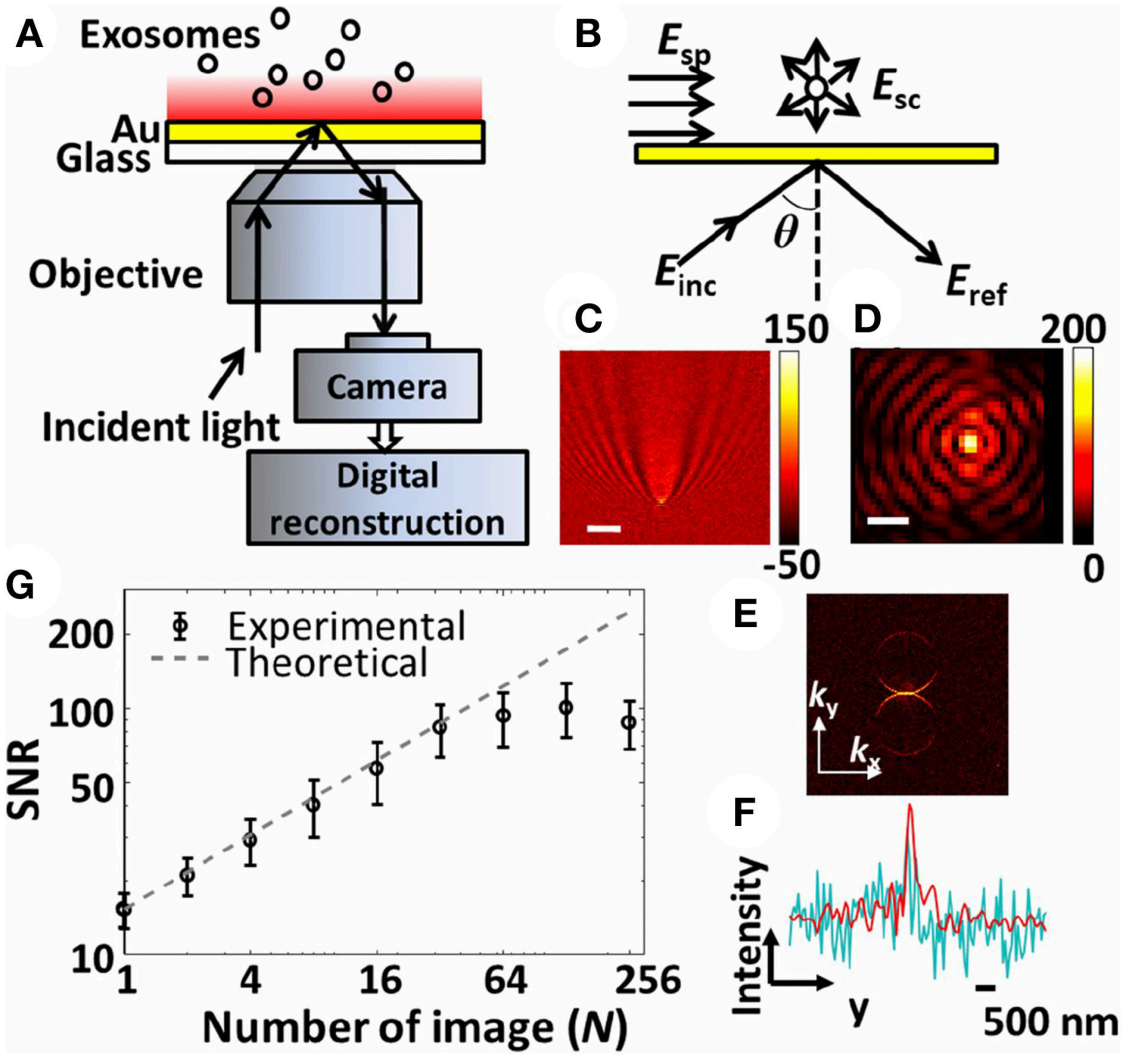
The interferometric plasmonic microscopy (iPM) system for physical (size) and biochemical (interaction) characterization of nEVs. **(A)** Schematic of the iPM. **(B)** Outline of the interferometric scattering model. **(C)** Image of 100 nm silica nanoparticle without (scale bar: 3 μm) and **(D)** with (scale bar: 300 nm) image reconstruction. **(E)** k-space image of C after 2D-FFT. **(F)** Longitudinal intensity profile across the silica particle in C (cyanine) and D (red). **(G)** Signal-to-noise profile in iPM detection of 100 nm silica particles using the running-average algorithm and the theoretical shot-noise limitation (dashed trace). Figure reproduced from Yang et al. (2018)© 2018.

*Other planar geometries*. A technique termed *interferometric plasmonic microscopy (iPM)* was recently introduced (Yang et al., 2018). The iPM sensors were glass cover slips coated with 2 nm Cr and 47 nm Au slabs. A CMOS camera and 637 nm laser were the central pieces of the optical setup (Figure 10). The compelling novelty was the common-path interferometry that harnesses the reflected light and scattering signal from objects within the reach of the evanescent field (~200 nm above the surface). The calibration of the system was carried out using silica nanoparticles. In-house image processing algorithms were employed to reconstitute the interaction events of A549 lung cancer cell line-derived nEVs. Different interaction scenarios were investigated, starting with nEVs and charged Au surfaces. As expected, adsorption of nEVs was dependent on the surface charge of the Au surface, e.g., only Au surfaces modified with HS-PEG-NH_2_ (positively charged surface) bound the anionic nEVs. When integrated with fluorescent microscopy and the nEV lipid membrane was labeled with DiIC18, fluorescence signals correlated well spatially with the iPM image analyses. Through the iPM analysis, nEVs demonstrated a continuous distribution between 30 and 150 nm with a peak value of ~62 nm. Notably, the NTA analyses showed higher values at ~120 nm. Although rigorous biophysical work was represented, the known EV/liposome shape reformation upon binding onto the Au surface was not addressed—which may contribute to the observed results.

**Figure 11:**
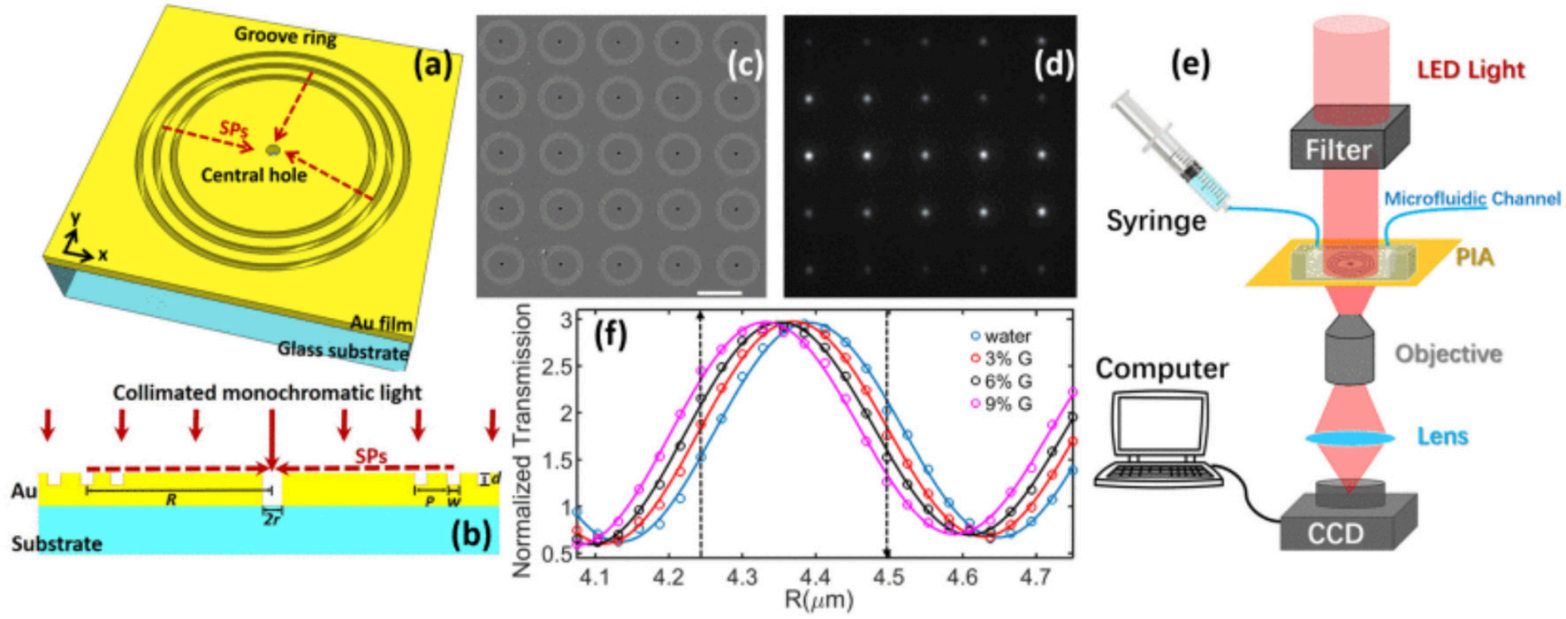
Plasmonic interferometer array (PIA) biochip. **(a)** Schematic of a ring-hole PIA and **(b)** cross-section view. **(c)** SEM image of a 5 × 5 array of plasmonic interferometer with increasing R from left to right, bottom to top. **(d)** Transmitted light can be imaged on a CCD camera. **(e)** Schematic diagram of the experimental setup. **(f)** Transmission of each interferometer was modulated sinusoidally by R, showing different sensitivity when glycerol-water solutions of increased concentrations were flowed on the sensor surface. Black arrows indicate the interferometers with highest sensitivities. Reprinted, with permission, from (Zeng et al., 2019) ©[2017] IEEE.

A similar nano-groove based sensor referred to as a *plasmonic interferometer array (PIA)* was invented for real-time detection of circulating nEV proteins with high sensitivity and portability (Zeng et al., 2019). The PIA biosensor measures intensity modulation at a single wavelength created by SP waves upon sensor illumination at the normal direction to artificial nano-grooves (Figure 11). SPs traveling along the radial direction of the rings interfere with light directly transmitted through a small aperture at the center of the grooves. Transmitted light intensity from each sensor is dependent on the change in RI between the opening and groove rings. To test feasibility of the PIA sensors, an 8 × 8 chessboard array was designed with two different radii (R1 = 4.25 μm and R2 = 4.5 μm). By increasing the RI, transmitted signal of R1 increases while R2 decreases, allowing for coupling of horizontally adjacent neighbors. The signal change was added by subtracting the decreasing R2 signal to the increasing R1 signal, which greatly reduced background noise and increased signal-to-noise ratio (SNR). To demonstrate the potential for early cancer diagnosis, EGFR (epidermal growth factor receptor) was used as a marker for A549 lung cancer cells. Following binding of anti-EGFR antibodies to the PIA biochip, high detection sensitivity was observed, with a SNR of 51.76, corresponding to a resolution of 3.86 × 10^8^ nEVs/mL. The PIA biochip was also integrated onto a smartphone-based camera microscope system, exhibiting SNR of 8.23 corresponding to a sensing resolution of 9.72 × 10^9^ nEVs/mL.

Self-assembled monolayer coated gold nanoislands (SAM-AuNIs) have been recently introduced as sensitive and inexpensive optochemical biosensors, produced by a two-step deposition-annealing procedure (Thakur et al., 2017). The authors included both nEVs and also larger MVs, isolated from four different sources (two *in vitro* cell lines, serum, and urine). Without surface functionalization, reported LOD of 0.194 μg/mL and linear dynamic range of 0.194–100 μg/mL were obtained. However, a concern is raised regarding isolation methods, which varied for each type of sample; cell culture supernatants employed UC and filtration, while serum and urine EVs were isolated using different commercial isolation reagents. The vaguely described MV isolation method stands out since the study essentially focuses on investigating nEVs vs. MVs, and the outcomes likely depend heavily on the chosen MV enrichment procedure. Also, given that each method likely yielded varying levels of purity, which were not directly assessed, non-EV originating contaminant proteins cannot be excluded and concentration estimates based on protein content may not be accurate. A critical suggestion would be to use a uniform isolation panel for all the different preparations and a consequent endeavor to qualitatively or quantitatively determine the purity of the preparations [further suggestions can be found e.g., in references: (Webber and Clayton, 2013; Maiolo et al., 2015)].

Another more recent LSPR sensor using AuNIs was reported, decorated with the peptide Venceremin (Vn96), which exhibits strong affinity to nEV surface heat-shock proteins (HSPs) overexpressed on many tumor cells (Bathini et al., 2018). Enthusiasm was limited by the heterogeneity of the AuNIs (20–80 nm), lack of clinical samples tested, and no assessment of statistical relevance (sensitivity, specificity, etc.). Joshi et al. introduced a very sensitive label-free LSPR biosensor to detect microRNA-10b (miR-10b) levels in biological fluids and lysed nEVs (Joshi et al., 2015). Single nucleotide specificity and attomolar (10^−18^ M) sensitivity was reported. Pancreatic cancer cells (PPCs) are characteristic to pancreatic ductal adenocarcinoma (PDAC), and PCCs are known to overexpress miR-10b in PDAC. The aim was to study whether PCCs release miR-10b into the cell culture medium or circulation, and to explore whether the miR-10b levels in nEVs could differentiate between chronic pancreatitis (CP), and individuals with and without PDAC pathology. Gold nanoprisms were synthesized, attached onto a glass substrate, and functionalized with complementary oligonucleotides. The developed LSPR sensor was demonstrated to work with PCCs (AsPC-1, BxPC-3, and PANC-1)-derived conditioned media and nEVs. Successful differentiation was also performed between PDAC or CP patients and normal controls in plasma, nEVs, and post-UC supernatants. The authors pinpoint various structural attributes in the LSPR sensors that enable high sensitivity, in particular the charge transport of the gold nanoprisms through the DNA backbone upon forming duplexes with the ssDNA. The regeneration capability and stability of the LSPR biosensors was also tested, and the authors concluded that the sensitivity remains unchanged at least over 5 days.

*Plasmonic nanoprobes*. A colorimetric nanoplasmonic LSPR assay has been introduced (Maiolo et al., 2015). In brief, when cationic AuNPs are titrated into contaminant-free nEVs, NPs cluster at the EV membrane to generate a LSPR red-shift that is proportional to the nEV concentration in the sample. If non-nEV soluble protein is present, the AuNPs are swarmed by a protein corona, preventing interplay with nEVs and no LSPR shift is present. Using this method, protein contaminants could be detected with LOD as low as 5 ng/μL. Notably, this method was also recently exploited in another study (Busatto et al., 2018) and may serve as a useful and rapid method to detect free protein contamination for general nEV characterization.

**Figure 12:**
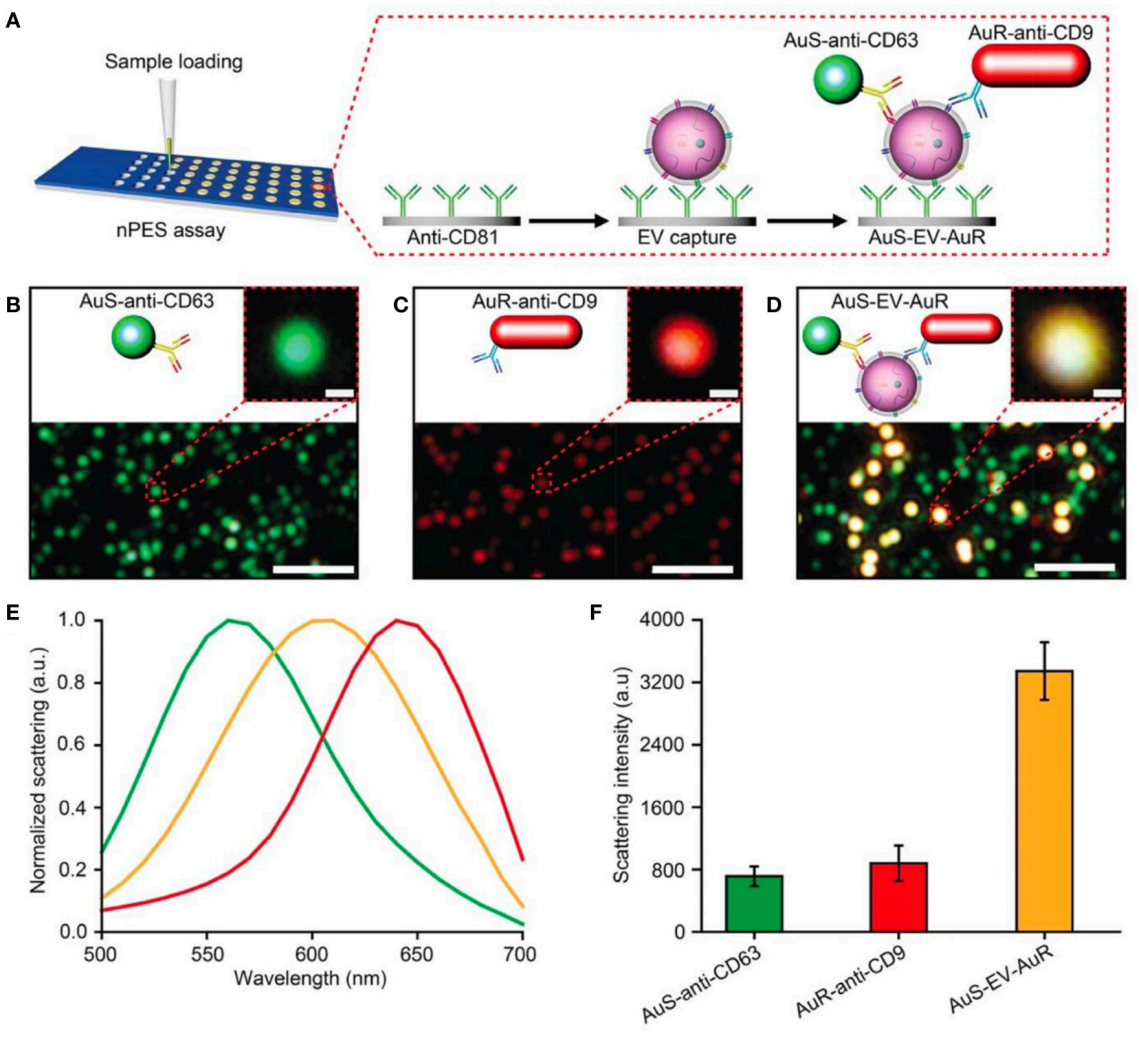
Design of an nPES platform for nEV detection. **(A)** Schematic overview of the nPES assay for specific detection of EVs. **(B–D)** Dark-field microscope (DFM) images of AuS-anti-CD63 (green), AuR-anti-CD9 (red) and AuS-EV-AuR complexes, which are detectable as bright yellow dots. Scale bars: main images, 2 μm; magnified images, 100 nm. **(E,F)** Scattering spectra **(E)** and intensities of AuS-anti-CD63 (ref. 48), AuR-anti-CD9, and AuS-EV-AuR complexes **(F)**. The scattering spectra and related intensities were recorded from 10 randomly selected particles for each complex by a spectrograph CCD equipped with a monochromator (CASCADE 512B, Roper Scientific). Data represent mean ± SEM; *n* = 10 replicates per sample. Reprinted by permission from Springer Nature (Liang et al., 2017) © 2017.

The *nano-plasmon enhanced scattering (nPES)* assay was developed and tested for pancreatic cancer as a model approach (Liang et al., 2017). A silica sensor chip comprising anti-CD81 functionalized wells was used to capture tumor-derived nEVs. The finding of the study was the concept of utilizing two types of plasmonically active Au nanoparticles, Au nanoparticles (AuNPs) and rods (AuNRs), to recognize tumor-derived nEVs. In the platform development phase, the authors noted that anti-CD63-AuNP and anti-CD9-AuNR nanoparticles formed AuNP-nEV-AuNR complexes on the sensor surface, and a significant spectral shift and intensity of the scattered light was observed (Figure 12). The nPES assay's performance was characterized by known nEV plasma concentrations, and nPES area ratios (area of nPES signal vs. well area) were used to evaluate unknown sample EV concentrations. The nPES assay outperformed ELISA in regard to sample consumption (1 μL vs. 150 μL), analysis time (5 h vs. > 24 h), sensitivity (0.2 vs. 77 ng/μL), and cost. After a meticulous proteomics and bioinformatics screening, the anti-CD63-AuNP probe was replaced with EphA2 (anti-EphA2-AuNP) as a pancreatic-cancer-specific EV marker. Cell line supernatants from normal (HPNE) and tumor (PANC-1) tissue at progressive tissue culture time points were analyzed to evaluate the specificity of the nPES platform. The PANC-1 EphA2 EV signals were significantly higher at all time points. As plasma samples from normal healthy controls (NC), pancreatitis, and pancreatic cancer patients were tested, the EphA2-EV signals were significantly higher. The same trend was perceived when a mouse xenograft model was used by injecting athymic nude mice with PANC-1 cells and analyzed for EphA2 blood levels every 10 days after the injection. The EphA2-EV levels also correlated with the tumor size. At the final part of the study the EphA2-EV nPES assay was harnessed to analyze a cohort of normal control individuals (*n* = 48), chronic pancreatitis (*n* = 48), and pancreatic cancer patients (*n* = 49) at different stages of the disease. The non-diagnostic biomarker CA19-9 (which is typically used to monitor the patient responses to therapy) was used for benchmarking the clinical performance of the EphA2-EV nPES levels. As 23 pancreatic cancer patients were evaluated pre and post neoadjuvant therapy, the EphA2-EV levels in comparison to CA19-9 more accurately reflected the treatment status for patients with good or partial therapy responses. The study was well outlined, and the results supported by additional experiments (SEM, WB) as well as carefully designed statistical analyses. As noted by the authors, the specificity and generalizability of the nPES platform could be improved further by replacing the secondary anti-CD9-AuNR probe with additional cancer-specific probes.

To summarize, LSPR techniques complement SPR methods well, and they can be used as stand-alone applications for nEVs. The needed instrumentation is relatively cost-effective and operator-friendly, simultaneously showing potential for miniaturization, automation and integration with e.g., microfluidics. A good example is the work by Maiolo et al. (2015), where the authors used a colorimetric LSPR assay able to infer the purity of nEV preparations by naked eye. However, cavities similar with SPR techniques can be found in the LSPR based nEV detection and characterization. While LSPR methods introduce compelling fundamental physics and theoretical viewpoints, the biological relevance with respect to specificity, sensitivity, and statistical power are often in need of addressing. Especially crude and complex sample matrices such as human plasma without any pre-treatment pose common issues. Many of these can be overcome e.g., meticulous experimental design, in-house technical innovations, biophysical modeling, and rigorous statistical calculations. Finally, LSPR particle heterogeneity is rarely accounted for, but certainly influences measurement to varying degrees, and should be reported.

### Surface Enhanced Raman Spectroscopy (SERS)

#### Physical Background of SERS

In Raman spectroscopy, laser light is used to irradiate a sample of interest and the resultant inelastically scattered photons are collected by a detector. The energies of such photons precisely correspond to the chemical bonds and structures present in the sample. Hence, a Raman spectrum consists of wavenumbers (inverse centimeters, cm^−1^) on the abscissa and scattering intensities on the ordinate. Raman spectroscopy affords high chemical specificity, minimal to no sample processing, is inherently non-destructive, and relatively inert to aqueous background. The main disadvantages for spontaneous Raman spectroscopy are weak signal and high fluorescent/photoluminescent backgrounds, particularly for biological specimens. However, these shortcomings can be efficiently overcome using SERS. Notably, even single molecule sensitivity can be achieved (Otto, 2002) as well as enhancement factors beyond ~10^15^. SERS is an inherently plasmonics-based technique, describing the exploitation of the coupling of photons to charge density oscillations (i.e., plasmons) of the conductive electrons in metals. Thus, in SERS, the high chemical specificity of Raman spectroscopy is combined with unprecedented sensitivity accomplished by plasmon-assisted scattering of molecules on or near (typically within ~10 nm) metal nanostructures. The formation of “hot spots”—where the highest signal enhancement is observed—is an important physical phenomenon, occurring where two or more SERS-active regions come into spatial contact, i.e., gap-mode plasmons (Figure 2). SERS is a powerful and sensitive modern analytical technology that has been successfully applied to medically-relevant detection of biomolecules like metabolites, nucleic acids, and proteins, and also to characterize and identify microorganisms and eukaryotic cells (Zheng et al., 2018). The high content of structural information, combined with sensitivity, quantification, and multiplexing opportunities, makes SERS a very powerful technique for the demanding purposes of nEV characterization.

#### Applications of SERS to nEVs

Vibrational spectroscopy without the assistance of plasmonics (i.e., Raman and infrared spectroscopy) has been applied to characterize nEVs and MVs in several cases, enabling quantitative measurement of global chemical composition analysis (e.g., relative amounts of nucleic acids, protein, lipids, sterols, etc.). Spontaneous Raman of both bulk EVs (Tatischeff et al., 2012; Krafft et al., 2016; Gualerzi et al., 2017) and also single vesicles trapped by optical tweezers (Smith et al., 2015; Carney et al., 2017; Lee et al., 2018; Kruglik et al., 2019) can be used to distinguish vesicles from various sources or disease contexts. Yet, spontaneous Raman spectroscopy has limited speed, and only relatively small numbers of EVs can be measured in a reasonable period, dramatically limiting its potential for clinical application. Therefore, it is more than likely necessary to exploit plasmonics enhancement of Raman scattering (i.e., SERS) to increase sensitivity and throughput.

##### SERS probes

**Figure 13:**
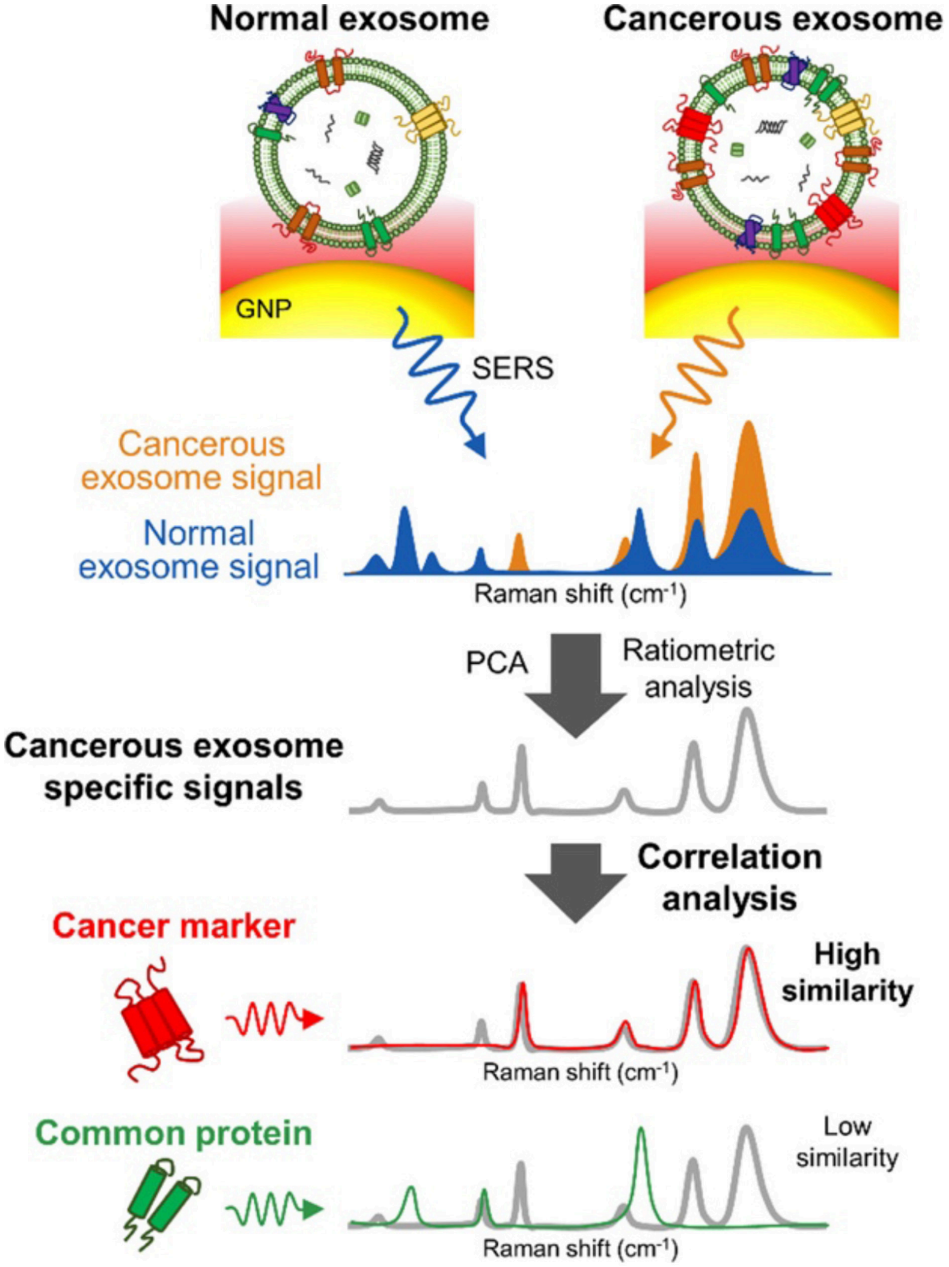
SERS detection of unique Raman scattering profiles. Lung cancer cell-derived and normal nEVs measured using AuNPs as plasmon-active signal amplifiers for SERS. Subsequent principal component analysis (PCA) can used to decompose acquired SERS spectra and perform correlation analysis against profiles of potential nEV surface protein markers. Cancerous exosome-specific protein markers are associated in terms of signal similarity. Reprinted with permission from Shin et al. (2018) Copyright (2018) American Chemical Society.

Several SERS studies using NP probes have been recently applied. Typically, the probes provide SERS enhancement of endogenous chemical nEV markers, such as lipids and membrane proteins, that vary amongst samples of interest (Figure 13). Park et al. applied 80 nm AuNPs to nEVs isolated by UC from two non-small-cell lung cancer (NSCLC) lines (H1299 and H522) in addition to normal alveolar cell line derived nEVs (Park et al., 2017a). Following deposition of AuNPs/nEVs onto a slide, PCA was used to classify resulting SERS spectra from 37 samples of H1299 nEVs, 34 samples of H522 nEVs, and 23 samples of alveolar nEVs into either NSCLC or normal groups with 95.3% sensitivity and 97.3% specificity. A variable loading plot for PCA was created with each point representing a unique wavenumber, allowing for greater classification sensitivity, down to 10^9^ particles/mL, rendering this technique more sensitive than chemiluminescence ELISA. Our own work examined the use of peptide-ligand decorated silver NPs (AgNPs) to capture and analyze nEVs binding α3ß1 integrin overexpressed on OvCa cell-derived nEVs, though should be extended to clinical samples for increased relevance (Lee et al., 2017).

Another study utilized a non-specific coating of 10 nm AuNPs to prepare surface-deposited nEVs for SERS interrogation (Stremersch et al., 2016). Here, nEVs were isolated by density gradient UC from either B16F10 melanoma cells or primary RBCs. First, cationic AuNPs were optimized to densely coat the anionic nEVs. DLS indicated ~800 AuNPs per B16F10 vesicle and ~1,200 per RBC vesicle. SERS signals were obtained for both samples and subjected to a partial least squares discriminant analysis (PLS-DA) and multivariate curve resolution alternating least squares (MCR-ALS). Both statistical models were calibrated with spectra from AuNPs alone and AuNPs with either B16F10 or RBC vesicles. Sensitivity was found to be 98.5, 88.0, and 95.1% and specificity was found to be 95.5, 95.4, and 98%, respectively. To demonstrate diagnostic potential, varying ratios of B16F10-derived and RBC-derived nEVs were mixed with AuNPs to provide a more accurate representation of an *in vivo* situation where tumor-associated vesicles are not the most abundant type. AuNP-coated nEVs from differing cell lines were able to be classified by biomolecular diversity by applying SERS technology in conjunction with PLS-DA statistical analysis, demonstrating the potential application of single vesicle identification for diagnostic use. Furthermore, throughput was high, with as many as 160,000 individual vesicles analyzed in a single day.

To increase chemical specificity beyond non-specific SERS probes, other approaches have incorporated immunolabeling techniques. One such study utilized a sandwich-based immunoassay (Zong et al., 2016), not unlike the geometry of a heterogeneous sandwich ELISA assay. In that study, two types of NPs were used: a SERS nanorod with a gold core and silver shell (Au@Ag NRs) and a magnetic nanobead, the latter synthesized by coating an iron oxide (Fe_3_O_4_) NP with a silica shell. Both Au@Ag NRs and magnetic nanobeads were further decorated with antibodies, enabling formation of a sandwich complex by capturing vesicles between the nanobeads and NRs. The resulting magnetic properties of the immunocomplexes permitted separation by magnet for SERS signal detection in collected precipitates. Using anti-CD63 and anti-HER2 antibody, nEVs secreted by SKBR3 could be readily distinguished from control MRC5 cell-derived nEVs. Decreasing concentrations of vesicles were immunoprecipitated to find an LOD of ~1,200 vesicles, with a total assay time of just 2 h. Another study incorporated three specific SERS probes decorated with aptamers as alternative biorecognition components to supplement antibodies (Wang et al., 2018). Here, silica coated magnetic beads were further coated with a plasmonic gold layer (MB@SiO_2_@Au) to be used as capturing substrates. CD63 surface protein aptamer was decorated onto the MB@SiO_2_@Au probes. In conjunction, AuNPs with complementary specific aptamers (AuNP@aptamer) were developed for use as a readout signal. Target nEVs were added to a mixture of the two probes to form a sandwich apta-immunocomplex. Similar to the previous study, the complexes could be precipitated by magnet for SERS readout. In this case, the signal became weaker with the addition of EVs that bind to the SERS probes while the signal from control non-specific probes remain unchanged. nEVs isolated using commercial kits from breast cancer cells (SKBR3), PCa cells (LNCaP), and colorectal cancer cells (T84) were each probed by various specific AuNP@aptamer complexes, using aptamers for H2, PSMA, and CEA, respectively. As the enhancements in various regions of SERS spectra varied widely between the samples, each presented a unique LOD: 32 nEVs per microliter for SKBR3, 203 nEVs per microliter for LNCaP, and 73 nEVs per microliter for T84. A similar study fabricated gold nanostars (AuNSs) “over-coated” with an outer gold film to trap Raman reporter molecules (4-MBA) between the layers (Tian et al., 2018). These AuNS@-4-MBA@Au probes were used as detection agents to probe nEVs pulled down on larger magnetic beads. Using a bivalent cholesterol-labeled DNA anchor to sandwich nEVs between the magnetic beads and plasmonic probes, reported LOD was low as 27 particles/μL. Ultimately, the use of commercial kits and small number of human clinical samples diminished our enthusiasm for each of the two previous studies, yet the proof-of-concept and low LODs are encouraging.

SERS probes have become a compelling alternative for plasmonic nEV detection and characterization. Virtually infinite amount of different nanofeatures can be crafted to provoke SERS signal enhancement followed by chemical functionalization with capturing and/or reporter compounds (Chinen et al., 2015; Liu et al., 2017; Zhang Y, et al., 2018). Three major aspects should however be more thoroughly addressed to make pioneering breakthroughs to the clinical settings. First, more rigorous testing with a large volume of clinical samples—even in proof-of-concept studies. Second, the development of well-validated and standardized SERS assay kits would be necessary for eventual adoption by non-experienced clinical operators. Third, Raman spectrometers in general are still typically too expensive and cumbersome for routine clinical analyses. Hand-held point-of-care spectrometers are already paving the way for these future diagnostic purposes.

##### SERS substrates

Ultimately, the use of nanoplasmonic probes may introduce some artifacts and complications, and samples are subjected to a number of undesirable mixing and washing steps. It is possible to realize SERS analysis using wholly label-free approaches with the use of active substrates, for example where plasmonic enhancement is afforded by the same material used to capture the biological agent of interest. Application of this concept to nEVs was introduced using super-hydrophobic surfaces (SHSs) in the form of patterned silicon micro-pillars decorated with silver nano-aggregates on the pillar top (Tirinato et al., 2012). The characteristic high hydrophobicity of these surfaces allows diluted solutions to concentrate a small number of molecules into a tiny region for analysis. Using commercial PEG isolation kits, nEVs were isolated from a colon tumor cell line (HCT116) and a healthy control cell line (CCD841-CoN) and diluted to a measured total protein concentration of ~0.2 ng/mL. Small aliquots of the mixture were dropped upon the SHSs and analyzed with SERS. Control nEVs showed higher intensities for peaks that corresponded to lipid vibrations while nEVs from the HCT116 tumor cell line showed larger SERS signals for protein and ribonucleic acid bases peaks. The differing intensities suggest that the two types of vesicles exhibit different biochemical composition, potentially due to differing cargo they carry. In another study, a large-scale SERS substrate was created from optical disk structures coated in varying levels of silver, allowing for tunable plasmonic resonances (Avella-Oliver et al., 2017). The biosensing capabilities of these substrates was shown by obtaining Raman spectra of hemoglobin and lung cancer cell line-derived nEVs, yet limit of detection, specificity, sensitivity, nor number of samples was reported. The authors suggest that prospective Raman microscope units can be based on optical disk drives to allow for transference of SERS to many environments. Another interesting SERS substrate was recently reported, featuring a nanohole array fabricated via electron beam lithography in both circular and square well form with pancreatic MSC EVs being introduced through drop casting. Following drying, Raman spectra were measured and compared, showing both shared spectral commonalities as well as variation between the EVs, the latter attributed to the presence or absence of proteins and nucleic acids. Again, however, no specificity, sensitivity, or number of samples analyzed was reported.

We also recently demonstrated application of a novel SERS substrate for nEV biochemical analysis (Lee et al., 2015). In our study, PDMS nanobowls coated with a thin (40 nm) silver film were used as SERS substrates set to capture small numbers of vesicles. nEVs were isolated from SKOV-3 cancer cell lines via both UC as well as with commercial TEIR for comparison. Twenty microliter of nEV dilutions could be dropped onto the SERS nanobowls and dried, pushing the vesicles into close contact with the substrate surface. SERS measurements were taken with TEIR kit solution itself, nEVs isolated via TEIR kit, and nEVs isolated via UC. Use of the nanobowls provided more reproducible SERS intensities because of more consistent “hot-spots” when compared to randomly aggregated nanoparticles used in other studies. A time-dependent analysis of the nEVs was performed, which showed that initially the vesicles maintain their form and the SERS signals represent the surfaces but as time progresses and the substrates dry out, the vesicles burst and spill their internal contents, causing a shift in the spectra. Thus, a distinction could be made between the surface and internal contents of the exosomes in solution. The LOD for this method was found to be one EV per nanobowl due to the limiting area created by the nanobowl dimensions.

**Figure 14:**
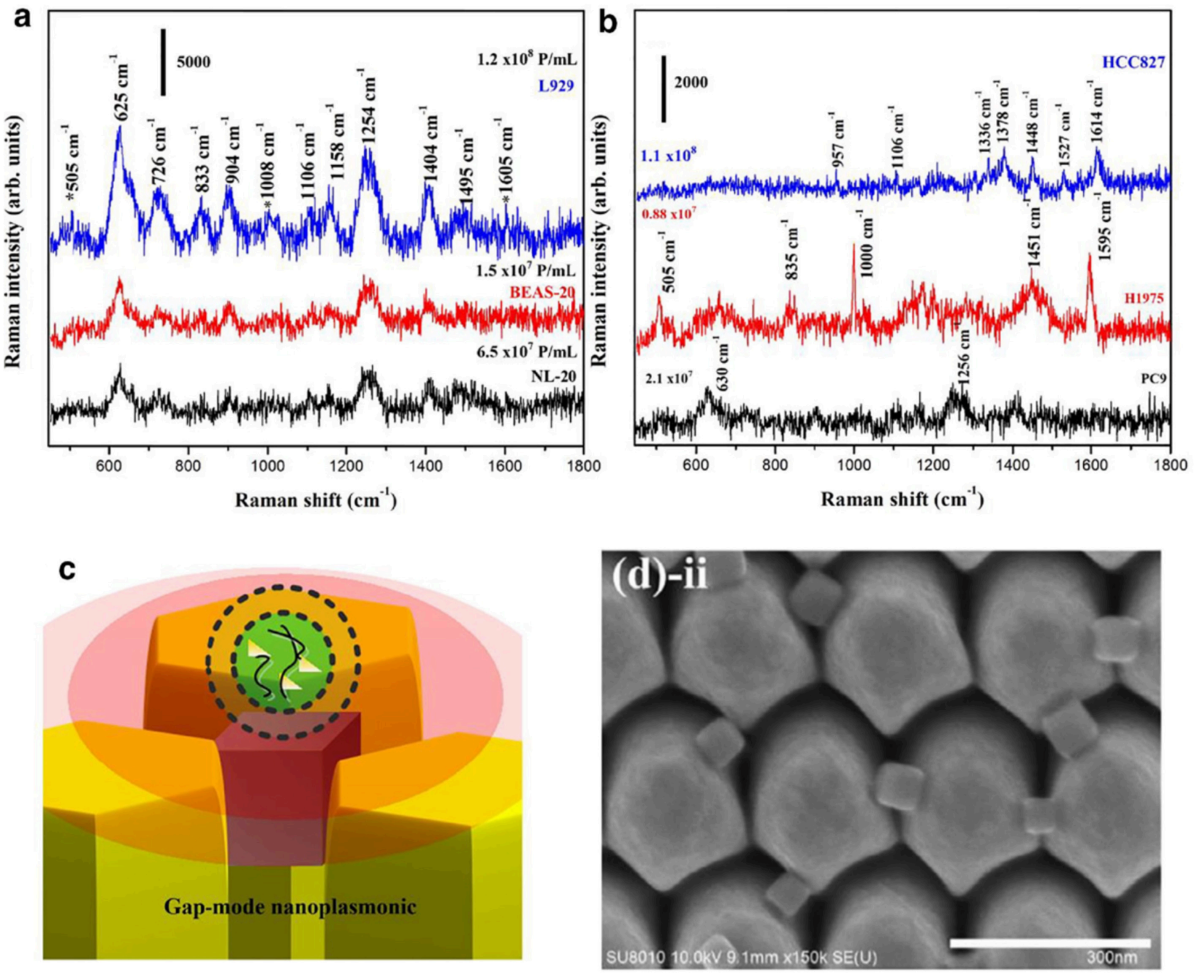
Plasmonic gap-mode SERS substrate SERS spectra of nEVs isolated from **(a)** normal lung cells and **(b)** lung cancer cells, recorded on an optimized Ag nanocubes on Au nanorod array (NCs-I-NRs). **(c)** A schematic of the showing the AgNCs embedded in the AuNR array the associated gap-mode electromagnetic enhancement for a bound nEV. **(d)** SEM images of the NCs-I-NRs from top-down perspective. Reprinted from Sivashanmugan et al. (2017) with permission from Elsevier.

A common design is to deposit AuNPs onto a slide to create a SERS substrate, for example using 10 nm AuNPs on a gold-coated slide that results in many hot-spot regions (Carmicheal et al., 2019). In that study, a cationic AuNP coating further facilitated binding of anionic nEVs, and SERS spectra from lipid and proteins distinguished pancreatic cancer serum samples from healthy controls in most patients with sensitivity and specificity of 90.6 and 97.1%, respectively. Shin et al. used antibody-coated 80 nm AuNPs in a similar fashion to detect characteristic SERS bands associated with nEV chemical content that distinguished NSCLC nEVs from healthy controls (Figure 13) (Shin et al., 2018). A more exotic SERS substrate was introduced by way of its strong plasmonic “gap-mode” (Sivashanmugan et al., 2017). Here, silver nanocubes (AgNCs) were assembled on an AuNR pillar array surface at varying AuNR tip-to-tip distances until an optimized geometry was found (Figure 14), i.e., resulting in the greatest amplification of SERS signal. The platform could distinguish nEVs derived from lung tumor cell lines (PC-9, H1975, and HCC827) compared with normal lung cell lines (NL-20, BEAS-2B, and L929). Titrations of nEVs were tested to show that detection was possible at concentrations 10^4^ to 10^5^ times lower than that found in typical blood samples.

While it is clear that SERS substrates with increasingly complex geometries could be of high impact for highly sensitive, minimally-invasive clinical diagnostics, there is a major drawback of the approach. It is not clear whether the intensity of SERS signals can be attributed to an increase in analyte concentration or rather that analyte is located at a more favorable position in the electromagnetic field or “hot spot,” thus quantitative measurements are far more difficult to obtain. New approaches will be needed to address this, e.g., via tunable site-specific binding to SERS probes and substrates. Another challenging aspect for this approach is to refine appropriate protocols for preparing substrate surfaces to ensure reproducible measurement, avoid non-specific absorption, and to attract EVs without the addition of bulky capture agents that interfere with the required spatial proximity of EV to surface.

## Future Outlook

### Applicability of Emerging Technologies

While this review provides a comprehensive summary on applications of nanoplasmonic techniques to analyze nEVs, we acknowledge that the journey is just beginning. Novel and creative approaches in the fields of nano and materials sciences, plasmonics, and optics will continue to improve nEV research. Even incremental modifications can be made to improve biosensor sensitivity in SPRS, such as by harnessing plasmonic coupling effects using plasmonic NPs (Špringer et al., 2014). Strongly absorbing dye molecules have also been used to enhance sensitivity and selectivity for an SPRS-based DNA hybridization assay (Granqvist et al., 2013), as have two-dimensional transition metal dichalcogenides (TMDCs) and graphene heterostructures (Zhao et al., 2018).

Many other emerging tools may have plasmonic applications as well. Surprisingly, IR spectroscopy has not gained a solid foothold amongst nEV characterization methods outside of a few notable examples (Mihály et al., 2017). As a future endeavor, surface enhanced infrared absorption spectroscopy (SEIRAS) could be exploited to amplify IR signal (Ataka and Heberle, 2007).

A nanomechanical resonator was recently demonstrated to weight the mass of single NPs down to 10 nm with attogram precision (Olcum et al., 2014). It is likely that select subpopulations could be identified using plasmonic adaptations. Oliveira-Rodrígues and co-workers developed a lateral flow immunoassay (LFIA) in a dipstick format using antibody-labeled AuNPs as probes (Oliveira-Rodríguez et al., 2016). Capillary forces induce a flow, the analytes of interest (e.g., nEVs) interact with the immobilized specimens and detection can be performed with a chosen probe. Being a cost-effective, fast, simple, and suitable for non-trained operators, such LFIA tests are especially promising for point-of-care (POC) applications. When higher throughput is desired, miniaturized lab-on-chip solutions with embedded microfluidics could be integrated. Indeed, the physical features of microfluidic techniques match very well the requirements of nEV research; negligible volumes (10^−9^ to 10^−18^ L) of liquids are being manipulated in channels with dimensions of tens to a few hundred microns. Furthermore, nEV analyses can be carried out in a short time, inexpensively, and isolation in addition to characterization is possible with high resolution and sensitivity. Microfluidics in combination with optics, nanoplasmonics and advanced data analysis tools offer an immense palette of opportunities for robust, automated, high-throughput and statistically relevant nEV research as well as moving from bench to bedside and commercial applications. We guide the reader to two excellent reviews that elucidate how modern microfluidic lab-on-chip techniques can be applied for nEV isolation, detection, and characterization (Gholizadeh et al., 2017; Chiriacò et al., 2018).

Still other emerging tools may be boosted by nanoplasmonic additions, such as the recent introduction of ZnO nanowire lateral displacement arrays for nEV fractionation and enrichment (Wunsch et al., 2016). This platform shares many features with the plasmonic nanoarrays presented above, and it is feasible that the physical separation obtained may be complimented by real-time chemical analysis afforded by incorporating plasmonic materials. Sensitivity of colorimetric approaches in general, for example using recent ZnO nanowire nEV “traps” could be improved using plasmonic probe detection (Chen et al., 2018). The recent advancements using graphene as a nanoplasmonic probe (de Abajo, 2014; Low and Avouris, 2014) has not yet extended to nEV detection, with one exception (Zhang P, et al., 2016). The emerging field of terahertz spectroscopy (Yang et al., 2016) has also only been applied to nEV analysis in one case (Knyazkova et al., 2018), yet also stands to greatly benefit with respect to sensitivity when combined with nanoplasmonic probes or surfaces (Gomez-Diaz et al., 2015). Finally, data analysis will be a critical area of research for nEVs, and we anticipate machine learning (or deep learning) to be a necessary tool to detangle the large swaths of data across patient samples afforded by emerging nanoplasmonic tools (Ballard et al., 2017; Malkiel et al., 2018). As spectroscopic and otherwise classifiable data collection rates increase, we expect these applications to rapidly grow in the years to come (Ko et al., 2017; Lee and Offerhaus, 2018).

### Critical Challenges

Diagnostic platforms for disease-associated nEVs have so far failed to achieve meaningful clinical translation, partly due to confusion and difficulties regarding their isolation and characterization, but also due to their low frequency in circulation compared to healthy background, particularly for early-stage patients. Nanoplasmonics offers the potential to realize the promise of exosomes and related nEVs to non-invasively report in real-time on the condition of cells and tissues. It is clear that nanoplasmonics-based approaches to detect and molecularly characterize nEVs offer spectacular signal enhancement compared to standard field-adopted methods. Yet, the majority of reported nanoplasmonic approaches are firmly ground at the level of proof-of-concept, displaying only incremental effects with limited scope. Although rational synthesis of plasmonic materials has been advancing tremendously in the past several years (Xi et al., 2018; Gurav et al., 2019), next-generation systems have not yet been realized and widespread clinical adoption is not feasible (Scarabelli, 2018). While plasmonics-based biosensing will certainly produce a huge impact on global health, particularly for low resource environments, there is still a long way to go. We summarize here several critical challenges with respect to nEVs (e.g., isolation, sampling, etc.) and engineering nanoplasmonic tools tailored for their study.

## Summary Points:

1. Researchers should focus on improving statistical power in clinically relevant sample sets, especially increasing number of patient samples, with the goal of high-power clinical trials.
2. Adherence to the ISEV suggested minimal information to characterize nEVs, along with careful reporting using the EV-TRACK database is highly advisable. Specifically, care should be taken to provide stringent chemical and physical characterization of EV preps using complementary field-validated methodology and protocols.
3. Development of materials engineering and chemical methods to improve the homogeneity of plasmonic probes and substrates is needed, the lack of which severely limits reproducibility and system stability or predictability. The effect of heterogeneity of nanoplasmonic probes and substrates is rarely considered and should be measured, reported, and the results contextualized accordingly.
4. Label-free approaches should be chosen when possible, in order to avoid known artifacts with respect to heterogeneous nEV labeling.
5. Plasmonic NP and substrate geometries should be tailored toward accounting for heterogeneity in size, a hallmark of nEVs. For example, nanohole arrays may be varied in hole diameter to accommodate a range of EV sizes, rather than a one-size-fits-all approach.
6. The vast majority of assays are currently limited to detecting transmembrane or lipid-bound biomolecules, based on immunoaffinity of whole nEVs to device surfaces. New assays should be developed to investigate and correlate intravesicular content, for example, plasmonic analysis before and after EV lysing.
7. Approaches capable of quantitative analysis, particularly toward single vesicle measurements, are critical to interpret and understand detailed structural and chemical information of nEVs in *in vivo* context. Diffraction-limited nanoplasmonic approaches may take advantage of patterning/spacing to address single or small groups of vesicles in their appropriate dimension range.

## Author Contributions

TR, BP, HK, and RC wrote and revised the manuscript and approved it for publication.

### Conflict of Interest Statement

The authors declare that the research was conducted in the absence of any commercial or financial relationships that could be construed as a potential conflict of interest.

## Abbreviations

Ab: antibody
AgNC: silver nanocube
AgNP: silver nanoparticle
AuNI: gold nanoisland
AuNP: gold nanoparticle
AuNR: gold nanorod
AuNS: gold nanostar
BCA: bicinchoninic acid
CTC: circulating tumor cell
DLS: dynamic light scattering
ELISA: enzyme-linked immunosorbent assay
EVs: extracellular vesicles
LOs: large oncosomes
LOD: limit of detection
LOQ: limit of quantification
LPPs: lipoprotein particles
LSPR: localized surface plasmon resonance
LTRS: laser trapping Raman spectroscopy
MP-SPR: multi-parametric SPR
MVs: microvesicles
nEVs: nanoscale extracellular vesicles
NTA: nanoparticle tracking analysis
OvCa: ovarian cancer
PIA: plasmonic interferometer array
PSA: prostate-specific antigen
RI: refractive index
RNP: ribonucleoproteins
SERS: surface enhanced Raman spectroscopy
SNR: signal-to-noise ratio
SPR: surface plasmon resonance
SPs: surface plasmons
SPPs: surface plasmon polaritons
TEIR: total exosome isolation reagent
TEM: transmission electron microscope
TIR: total internal reflection
UF: ultrafiltration
WB: Western blot.

## References

[B1] Aatonen M. T. Ohman T. Nyman T. A. Laitinen S. Grönholm H. Siljander P.R. (2014). Isolation and characterization of platelet-derived extracellular vesicles. J. Extracell. Vesic. 3:3791. 10.3402/jev.v3.24692 25147646 PMC4125723

[B2] Aguado B. A. Bushnell G. G. Rao S. S. Jeruss J. S. Shea L.D. (2017). Engineering the pre-metastatic niche. Nat. Biomed. Eng. 1:0077. 10.1038/s41551-017-0077 28989814 PMC5628747

[B3] Andreu Z. Rivas E. Sanguino-Pascual A. Lamana A. Marazuela M. González-Alvaro I.. (2016). Comparative analysis of EV isolation procedures for miRNAs detection in serum samples. J. Extracell. Vesic. 5:31655. 10.3402/jev.v5.31655 27330048 PMC4916259

[B4] Anker J. N. Hall W. P. Lyandres O. Shah N. C. Zhao J. Van Duyne R.P. (2008). Biosensing with plasmonic nanosensors. Nat. Mater. 7, 442–453. 10.1038/nmat2162 18497851

[B5] Arraud N. Gounou C. Turpin D. Brisson A.R. (2015). Fluorescence triggering: a general strategy for enumerating and phenotyping extracellular vesicles by flow cytometry. Cytometry 89, 184–195. 10.1002/cyto.a.22669 25857288

[B6] Ataka K. Heberle J. (2007). Biochemical applications of surface-enhanced infrared absorption spectroscopy. Anal. Bioanal. Chem. 388, 47–54. 10.1007/s00216-006-1071-4 17242890 PMC1839866

[B7] Avella-Oliver M. Puchades R. Wachsmann-Hogiu S. Maquieiera A. (2017). Label-free SERS analysis of proteins and exosomes with large-scale substrates from recordable compact disks. Sens. Actuat. Chem. 252, 657–662. 10.1016/j.snb.2017.06.058

[B8] Ballard Z. S. Shir D. Bhardwaj A. Bazargan S. Sathianathan S.. (2017). Computational sensing using low-cost and mobile plasmonic readers designed by machine learning. ACS Nano 11, 2266–2274. 10.1021/acsnano.7b00105 28128933 PMC5451292

[B9] Barile L. Vassalli G. (2017). Exosomes: therapy delivery tools and biomarkers of diseases. Pharmacol. Ther. 174, 63–78. 10.1016/j.pharmthera.2017.02.020 28202367

[B10] Bathini S. Badilescu S. Ouellette R. Ghosh A. Packirisamy M. Raju D. (2018). Nano-bio interactions of extracellular vesicles with gold nanoislands for early cancer diagnosis. Research 2018:3917986 10.1155/2018/3917986 PMC6750071 31549028

[B11] Blanc L. Vidal M. (2017). New insights into the function of Rab GTPases in the context of exosomal secretion. Small GTPases 9, 95–106. 10.1080/21541248.2016.1264352 28135905 PMC5902209

[B12] Bobrie A. Théry C. (2013). Exosomes and communication between tumours and the immune system: are all exosomes equal? Biochem. Soc. Trans. 41, 263–267. 10.1042/BST20120245 23356294

[B13] Busatto S. Giacomini A. Montis C. Ronca R. Bergese P. (2018). Uptake profiles of human serum exosomes by murine and human tumor cells through combined use of colloidal nanoplasmonics and flow cytofluorimetric analysis. Anal. Chem. 90, 7855–7861. 10.1021/acs.analchem.7b04374 29870225

[B14] Buzas E. I. György B. Nagy G. Falus A. Gay S. (2014). Emerging role of extracellular vesicles in inflammatory diseases. Nat. Rev. Rheumatol. 10, 356–364. 10.1038/nrrheum.2014.19 24535546

[B15] Campbell C. T. Kim G. (2007). SPR microscopy and its applications to high-throughput analyses of biomolecular binding events and their kinetics. Biomaterials 28, 2380–2392. 10.1016/j.biomaterials.2007.01.047 17337300

[B16] Carmicheal J. Hayashi C. Huang X. Liu L. Lu Y. Krasnoslobodtsev A. (2019). Label-free characterization of exosome via surface enhanced Raman spectroscopy for the early detection of pancreatic Cancer. Nanomed. Nanotechnol. Biol. Med. 90, 88–96. 10.1016/j.nano.2018.11.008 PMC6532067 30550805

[B17] Carney R. P. Hazari S. Colquhoun M. Tran D. Hwang B. Mulligan M. S.. (2017). Multispectral optical tweezers for biochemical fingerprinting of CD9-positive exosome subpopulations. Anal. Chem. 89, 5357–5363. 10.1021/acs.analchem.7b00017 28345878 PMC5551404

[B18] Chen H. Kou X. Yang Z. Ni W. Wang J. (2008). Shape- and size-dependent refractive index sensitivity of gold nanoparticles. Anal. Chem. 24, 5233–5237. 10.1021/la800305j 18435552

[B19] Chen X. Parker S. G. Zou G. Su W. Zhang Q. (2010). β-Cyclodextrin-functionalized silver nanoparticles for the naked eye detection of aromatic isomers. ACS Nano 4, 6387–6394. 10.1021/nn1016605 20973513

[B20] Chen Z. Cheng S. B. Cao P. Qui Q. F. Chen Y. Xie M.. (2018). Detection of exosomes by ZnO nanowires coated three-dimensional scaffold chip device. Biosens. Bioelectr. 122, 211–216. 10.1016/j.bios.2018.09.033 30265971

[B21] Chinen A. B. Guan C. M. Ferrer J. R. Barnaby S. N. Merkel T. J. Mirkin C. A. (2015). Nanoparticle probes for the detection of cancer biomarkers, cells, and tissues by fluorescence. Chem. Rev. 115, 10530–10574. 10.1021/acs.chemrev.5b00321 26313138 PMC5457709

[B22] Chiriacò M. S. Bianco M. Nigro A. Primiceri E. Ferrara F. Romano A.. (2018). Lab-on-chip for exosomes and microvesicles detection and characterization. Sensors 18:3175. 10.3390/s18103175 30241303 PMC6210978

[B23] Cocucci E. Meldolesi J. (2015). Ectosomes and exosomes: shedding the confusion between extracellular vesicles. Trends Cell. Biol. 25, 364–372. 10.1016/j.tcb.2015.01.004 25683921

[B24] Coleman B. M. Hill A.F. (2015). Extracellular vesicles - their role in the packaging and spread of misfolded proteins associated with neurodegenerative diseases. Semin. Cell Dev. Biol. 40, 89–96. 10.1016/j.semcdb.2015.02.007 25704308

[B25] Consortium E.-T. Van Deun J. Mestdagh P. Agostinis P. Akay Ö. Anand S. (2017). EV-TRACK: transparent reporting and centralizing knowledge in extracellular vesicle research. Nat. Methods 14, 228–232. 10.1038/nmeth.4185 28245209

[B26] Costa-Silva B. Aiello N. M. Ocean A. J. Singh S. Zhang H. Thakur B. K.. (2015). Pancreatic cancer exosomes initiate pre-metastatic niche formation in the liver. Nat. Cell Biol. 17, 816–826. 10.1038/ncb3169 25985394 PMC5769922

[B27] Coumans F. A. W. Brisson A. R. Buzas E. I. Dignat-George F. Drees E. E. E. El-Andaloussi S.. (2017). Methodological guidelines to study extracellular vesicles. Circ. Res. 120, 1632–1648. 10.1161/CIRCRESAHA.117.309417 28495994

[B28] Daaboul G. G. Gagni P. Benussi L. Bettotti P. Ciani M. Cretich M. (2016). Digital detection of exosomes by interferometric imaging. Sci. Rep. 2016:37246 10.1038/srep37246 PMC5112555 27853258

[B29] Dauros Singorenko P. Chang V. Whitcombe A. Simonov D. Hong J. Phillips A.. (2017). Isolation of membrane vesicles from prokaryotes: a technical and biological comparison reveals heterogeneity. J. Extracell. Vesic. 6:1324731. 10.1080/20013078.2017.1324731 28717421 PMC5505020

[B30] de Abajo F. J. G. (2014). Graphene plasmonics: challenges and opportunities. ACS Phot. 1, 135–152. 10.1021/ph400147y

[B31] de la Rica R. Stevens M. M. (2012). Plasmonic ELISA for the ultrasensitive detection of disease biomarkers with the naked eye. Nat. Nanotechnol. 7, 821–824. 10.1038/nnano.2012.186 23103935

[B32] de Rond L. van der Pol E. Hau C. M. Varga Z. Sturk A. van Leeuwen T. G.. (2018). Comparison of generic fluorescent markers for detection of extracellular vesicles by flow cytometry. Clin. Chem. 64, 680–689. 10.1373/clinchem.2017.278978 29453194

[B33] Deatherage B. L. Cookson B. T. (2012). Membrane vesicle release in bacteria, eukaryotes, and archaea: a conserved yet underappreciated aspect of microbial life. Infect. Immun. 80, 1948–1957. 10.1128/IAI.06014-11 22409932 PMC3370574

[B34] Di Noto G. Bugatti A. Zendrini A. Mazzoldi E. L. Montanelli A. Caimi L.. (2016). Merging colloidal nanoplasmonics and surface plasmon resonance spectroscopy for enhanced profiling of multiple myeloma-derived exosomes. Biosens. Bioelectr. 77, 518–524. 10.1016/j.bios.2015.09.061 26469728

[B35] Di Noto G. Chiarini M. Paolini L. Mazzoldi E. L. Giustini V. Radeghieri A. (2014). Immunoglobulin free light chains and GAGs mediate multiple myeloma extracellular vesicles uptake and secondary NfkB nuclear translocation. Front. Immunol. 5:654 10.3389/fimmu.2014.00517 25386176 PMC4209816

[B36] Ding M. Wang C. Lu X. Zhang C. Zhou Z. Chen X.. (2018). Comparison of commercial exosome isolation kits for circulating exosomal microRNA profiling. Anal. Bioanal. Chem. 410, 3805–3814. 10.1007/s00216-018-1052-4 29671027

[B37] Ekgasit S. Thammacharoen C. Knoll W. (2004). Surface plasmon resonance spectroscopy based on evanescent field treatment. Anal. Chem. 76, 561–568. 10.1021/ac035042v 14750847

[B38] Elghanian R. Storhoff J. J. Mucic R. C. Letsinger R. L. Mirkin C.A. (1997). Selective colorimetric detection of polynucleotides based on the distance-dependent optical properties of gold nanoparticles. Science 277, 1078–1081. 10.1126/science.277.5329.1078 9262471

[B39] Erdbrügger U. Lannigan J. (2016). Analytical challenges of extracellular vesicle detection: a comparison of different techniques. Cytomet. Part A 89, 123–134. 10.1002/cyto.a.22795 26651033

[B40] Ferhan A. R. Jackman J. A. Park J. H. Cho N. J. Kim D.H. (2018). Nanoplasmonic sensors for detecting circulating cancer biomarkers. Adv. Drug Deliv. Rev. 125, 48–77. 10.1016/j.addr.2017.12.004 29247763

[B41] Ferhan A. R. Kim D.-H. (2016). Nanoparticle polymer composites on solid substrates for plasmonic sensing applications. Nano Today 11, 415–434. 10.1016/j.nantod.2016.07.001

[B42] Gholizadeh S. Shehata Draz M. Zarghooni M. Sanati-Nezhad A. Ghavami S. Shafiee H.. (2017). Microfluidic approaches for isolation, detection, and characterization of extracellular vesicles: current status and future directions. Biosens. Bioelectr. 91, 588–605. 10.1016/j.bios.2016.12.062 28088752 PMC5323331

[B43] Gomez-Diaz J. S. Moldovan C. Capdevila S. Romeu J. Bernard L. S. Magrez A.. (2015). Self-biased reconfigurable graphene stacks for terahertz plasmonics. Nat. Commun. 6:6334. 10.1038/ncomms7334 25727797

[B44] Gould S. J. Booth A. M. Hildreth J. E. (2011). The Trojan exosome hypothesis. Proc. Natl. Acad. Sci. U.S.A. 100, 10592–10597. 10.1073/pnas.1831413100 12947040 PMC196848

[B45] Gould S. J. Raposo G. (2013). As we wait: coping with an imperfect nomenclature for extracellular vesicles. J. Extracell. Vesic. 2:20389 10.3402/jev.v2i0.20389 24009890 PMC3760635

[B46] Granqvist N. Hanning A. Eng L. Tuppurainen J. Viitala T. (2013). Label-enhanced surface plasmon resonance: a new concept for improved performance in optical biosensor analysis. Sensors 13, 15348–15363. 10.3390/s131115348 24217357 PMC3871110

[B47] Grasso L. Wyss R. Weidenauer L. Thampi A. Demurtas D. Prudent M.. (2015). Molecular screening of cancer-derived exosomes by surface plasmon resonance spectroscopy. Anal. Bioanal. Chem. 407, 5425–5432. 10.1007/s00216-015-8711-5 25925862 PMC4477949

[B48] Gualerzi A. Niada S. Giannasi C. Picciolini S. Morasso C. Vanna R.. (2017). Raman spectroscopy uncovers biochemical tissue-related features of extracellular vesicles from mesenchymal stromal cells. Sci. Rep. 7:9820. 10.1038/s41598-017-10448-1 28852131 PMC5575260

[B49] Guo L. Chen G. Kim D. H. (2010). Three-dimensionally assembled gold nanostructures for plasmonic biosensors. Anal. Chem. 82, 5147–5153. 10.1021/ac100346z 20469841

[B50] Gurav D. D. Jia Y. Ye J. Qian K. (2019). Design of plasmonic nanomaterials for diagnostic spectrometry. Nanosc. Adv. 356, 459–469. 10.1039/C8NA00319J PMC9473262 36132258

[B51] Haes A. J. Van Duyne R. P. (2004). A unified view of propagating and localized surface plasmon resonance biosensors. Anal. Bioanal. Chem. 379, 920–930. 10.1007/s00216-004-2708-9 15338088

[B52] Haes A. J. Zou S. Schatz G. C. Van Duyne R. P. (2004). A nanoscale optical biosensor: the long range distance dependence of the localized surface plasmon resonance of noble metal nanoparticles. J. Phys. Chem. B 108, 109–116. 10.1021/jp0361327

[B53] Hall W. P. Ngatia S. N. Van Duyne R. P. (2011). LSPR biosensor signal enhancement using nanoparticle-antibody conjugates. J. Phys. Chem. C 115, 1410–1414. 10.1021/jp106912p 21660207 PMC3109750

[B54] Hayt W. H. Buck J. A. (2001). Engineering Electromagnetics, 6th Edn. Atlanta, GA: The McGraw Companies.

[B55] Hermann R. J. Gordon M. J. (2018). Nanoscale optical microscopy and spectroscopy using near-field probes. Annu. Rev. Chem. Biomol. Eng. 9, 365–387. 10.1146/annurev-chembioeng-060817-084150 29596000

[B56] Hill R. T. (2015). Plasmonic biosensors. Wiley Interdiscipl. Rev. Nanomed. Nanobiotechnol. 7, 152–168. 10.1002/wnan.1314 25377594 PMC4308478

[B57] Ho F. H. Wu Y. H. Ujihara M. Imae T. (2012). A solution-based nano-plasmonic sensing technique by using gold nanorods. Analyst 137, 2545–2548. 10.1039/c2an35101c 22479700

[B58] Hoshino A. Costa-Silva B. Shen T. L. Rodrigues G. Hashimoto A. Tesic Mark M.. (2015). Tumour exosome integrins determine organotropic metastasis. Nature 527, 329–335. 10.1038/nature15756 26524530 PMC4788391

[B59] Hosseinkhani B. van den Akker N. D'Haen J. Gagliardi M. Struys T. Lambrichts I.. (2017). Direct detection of nano-scale extracellular vesicles derived from inflammation-triggered endothelial cells using surface plasmon resonance. Nanomed. Nanotechnol. Biol. Med. 13, 1663–1671. 10.1016/j.nano.2017.03.010 28366819

[B60] Im H. Shao H. Park Y. L. Peterson V. M. Castro C. M. Weissleder R.. (2014). Label-free detection and molecular profiling of exosomes with a nano-plasmonic sensor. Nat. Biotechnol. 32, 490–495. 10.1038/nbt.2886 24752081 PMC4356947

[B61] Im H. Shao H. Weissleder R. Castro C. M. Lee H. (2015). Nano-plasmonic exosome diagnostics. Expert Rev. Mol. Diagn. 15, 725–733. 10.1586/14737159.2015.1041378 25936957 PMC4675626

[B62] Jackman J. A. Ferhan A. R. Cho N. J. (2017). Nanoplasmonic sensors for biointerfacial science. Chem. Soc. Rev. 46, 3615–3660. 10.1039/C6CS00494F 28383083

[B63] Jakobsen K. R. Paulsen B. S. Bæk R. Varming K. Sorensen B. S. Jørgensen M. M. (2015). Exosomal proteins as potential diagnostic markers in advanced non-small cell lung carcinoma. J. Extracell. Vesicles 4:26659. 10.3402/jev.v4.26659 25735706 PMC4348413

[B64] Janas A. M. Sapo,n K. Janas T. Stowell M. H. Janas T. (2016). Exosomes and other extracellular vesicles in neural cells and neurodegenerative diseases. Biochim. Biophys. Acta 1858, 1139–1151. 10.1016/j.bbamem.2016.02.011 26874206

[B65] Jans H. Huo Q. (2012). Gold nanoparticle-enabled biological and chemical detection and analysis. Chem. Soc. Rev. 41, 2849–2866. 10.1039/C1CS15280G 22182959

[B66] Jeyaram A. Jay S. M. (2018). Preservation and storage stability of extracellular vesicles for therapeutic applications. AAPS J. 20:1. 10.1208/s12248-017-0160-y 29181730 PMC6582961

[B67] Jing H. He X. Zheng J. (2018). Exosomes and regenerative medicine: state of the art and perspectives. Translat. Res. 196, 1–16. 10.1016/j.trsl.2018.01.005 29432720

[B68] Johnstone R. M. Adam M. Hammond J. R. Orr L. Turbide C. (1987). Vesicle formation during reticulocyte maturation. Association of plasma membrane activities with released vesicles (exosomes). J. Biol. Chem. 262, 9412–9420. 3597417

[B69] Joshi G. K. Deitz-McElyea S. Liyanage T. Lawrence K. Mali S. Sardar R.. (2015). Label-free nanoplasmonic-based short noncoding RNA sensing at attomolar concentrations allows for quantitative and highly specific assay of microRNA-10b in biological fluids and circulating exosomes. ACS Nano. 9, 11075–11089. 10.1021/acsnano.5b04527 26444644 PMC4660391

[B70] Knyazkova A. I. Yunusova N. V. Tugutova E. A. Ilyasova E. E. Sandykova E. A. Zasedatel V. S. (2018). THz laser spectroscopy exosome analysis of saliva and blood plasma, in International Conference on Atomic and Molecular Pulsed Lasers XIII (Tomsk: International Society for Optics and Photonics).

[B71] Ko J. Bhagwat N. Yee S. S. Ortiz N. Sahmoud A. Black T.. (2017). Combining machine learning and nanofluidic technology to diagnose pancreatic cancer using exosomes. ACS Nano 11, 11182–11193. 10.1021/acsnano.7b05503 29019651

[B72] Koenen R. R. Aikawa E. (2018). Editorial: extracellular vesicle-mediated processes in cardiovascular diseases. Front. Cardiovasc. Med. 5, 1–2. 10.3389/fcvm.2018.00133 30283791 PMC6157324

[B73] Konoshenko M. Y. Lekchnov E. A. Vlassov A. V. Laktionov P. P. (2018). Isolation of extracellular vesicles: general methodologies and latest trends. Biomed. Res. Int. 2018, 1–27. 10.1155/2018/8545347 29662902 PMC5831698

[B74] Krafft C. Wilhelm K. Eremin A. Nestel S. von Bubnoff N. Schultze-Seemann W.. (2016). A specific spectral signature of serum and plasma-derived extracellular vesicles for cancer screening. Nanomed. Nanotechnol. Biol. Med. 13, 835–841. 10.1016/j.nano.2016.11.016 27965168

[B75] Kretschmann E. Raether H. (1968). Notizen: radiative decay of non radiative surface plasmons excited by light. Zeitschrift Naturforschung 23, 2135–2136. 10.1515/zna-1968-1247

[B76] Kruglik S. G. Royo F. Guigner J. M. Palomo L. Seksek O. Turpin P. Y.. (2019). Raman tweezers microspectroscopy of circa 100 nm extracellular vesicles. Nanoscale 11, 1661–1679. 10.1039/C8NR04677H 30620023

[B77] Kusuma G. D. Barabadi M. Tan J. L. Morton D. A. V. Frith J. E. Lim R. (2018). To protect and to preserve: novel preservation strategies for extracellular vesicles. Front. Pharmacol. 9:1199. 10.3389/fphar.2018.01199 30420804 PMC6215815

[B78] Lacroix R. Judicone C. Poncelet P. Robert S. Arnaud L. Sampol J.. (2012). Impact of pre-analytical parameters on the measurement of circulating microparticles: towards standardization of protocol. J. Thromb. Haemost. 10, 437–446. 10.1111/j.1538-7836.2011.04610.x 22212198

[B79] Lai X. Wang M. McElyea S. D. Sherman S. House M. Korc M. (2017). A microRNA signature in circulating exosomes is superior to exosomal glypican-1 levels for diagnosing pancreatic cancer. Cancer Lett. 393, 86–93. 10.1016/j.canlet.2017.02.019 28232049 PMC5386003

[B80] Lee C. Carney R. P. Hazari S. Smith Z. J. Knudson A. Robertson C. S.. (2015). 3D plasmonic nanobowl platform for the study of exosomes in solution. Nanoscale 7, 9290–9297. 10.1039/C5NR01333J 25939587 PMC11781986

[B81] Lee C. Carney R. P. Lam K. S. Chan J. W. (2017). SERS analysis of selectively captured exosomes using an integrin-specific peptide ligand. J. Raman Spectrosc. 48, 1771–1776. 10.1002/jrs.5234

[B82] Lee W. Nanou A. Rikkert L. Coumans F. A. W. Otto C. Terstappen L. W. M. M.. (2018). Label-free prostate cancer detection by characterization of extracellular vesicles using raman spectroscope. Anal. Chem. 90, 11290–11296. 10.1021/acs.analchem.8b01831 30157378 PMC6170952

[B83] Lee W. Offerhaus H. L. (2018). Classifying Raman spectra of extracellular vesicles using a convolutional neural network, in XXVI International Conference on Raman Spectroscopy (Jeju).

[B84] Liang K. Liu F. Fan J. Sun D. Liu C. Lyon C. J. (2017). Nanoplasmonic quantification of tumour-derived extracellular vesicles in plasma microsamples for diagnosis and treatment monitoring. Nat. Biomed. Eng. 1:0021 10.1038/s41551-016-0021 28791195 PMC5543996

[B85] Liu A. Wang G. Wang F. Zhang Y. (2017). Gold nanostructures with near-infrared plasmonic resonance: synthesis and surface functionalization. Coord. Chem. Rev. 336, 28–42. 10.1016/j.ccr.2016.12.019

[B86] Lobb R. J. Becker M. Wen S. W. Wong C. S. Wiegmans A. P. Leimgruber A.. (2015). Optimized exosome isolation protocol for cell culture supernatant and human plasma. J. Extracell. Vesicles 4:27031. 10.3402/jev.v4.27031 26194179 PMC4507751

[B87] Lopez G. A. Estevez M.-C. Soler M. Lechuga L. M. (2016). Recent advances in nanoplasmonic biosensors: applications and lab-on-a-chip integration. Nanophotonics 6, 123–136. 10.1515/nanoph-2016-0101

[B88] Lötvall J. Hill A. F. Hochberg F. Buzás E. I. Di Vizio D. Gardiner C.. (2014). Minimal experimental requirements for definition of extracellular vesicles and their functions: a position statement from the International Society for Extracellular Vesicles. J. Extracell. Vesicles 3:26913. 10.3402/jev.v3.26913 25536934 PMC4275645

[B89] Low T. Avouris P. (2014). Graphene plasmonics for terahertz to mid-infrared applications. ACS Nano 8, 1086–1101. 10.1021/nn406627u 24484181

[B90] Maier S. A. (2007). Plasmonics: Fundamentals and Applications. New York, NY: Springer 10.1007/0-387-37825-1

[B91] Maiolo D. Paolini L. Di Noto G. Zendrini A. Berti D. Bergese P.. (2015). Colorimetric nanoplasmonic assay to determine purity and titrate extracellular vesicles. Anal. Chem. 87, 4168–4176. 10.1021/ac504861d 25674701

[B92] Malkiel I. Mrejen M. Nagler A. Arieli U. Wolf L. Suchowski H. (2018). Plasmonic nanostructure design and characterization via deep learning. Light Sci. Appl. 7:60. 10.1038/s41377-018-0060-7 30863544 PMC6123479

[B93] Mathieu M. Martin-Jaular L. Lavieu G. Théry C. (2019). Specificities of secretion and uptake of exosomes and other extracellular vesicles for cell-to-cell communication. Nat. Cell Biol. 21, 9–17. 10.1038/s41556-018-0250-9 30602770

[B94] McLeod E. Dincer T. U. Veli M. Ertas Y. N. Nguyen C. Luo W.. (2015). High-throughput and label-free single nanoparticle sizing based on time-resolved on-chip microscopy. ACS Nano 9, 3265–3273. 10.1021/acsnano.5b00388 25688665

[B95] Meckes D. G. (2015). Exosomal communication goes viral. J. Virol. 89, 5200–5203. 10.1128/JVI.02470-14 25740980 PMC4442506

[B96] Melo S. A. Sugimoto H. O'Connell J. T. Kato N. Villanueva A. Vidal A.. (2014). Cancer exosomes perform cell-independent microRNA biogenesis and promote tumorigenesis. Cancer Cell, 26, 707–721. 10.1016/j.ccell.2014.09.005 25446899 PMC4254633

[B97] Michel D. Xiao F. Alameh K. (2017). A compact, flexible fiber-optic surface plasmon resonance sensor with changeable sensor chips. Sens. Actuat. Chem. 246, 258–261. 10.1016/j.snb.2017.02.064

[B98] Mihály J. Deák R. Szigyártó I. C. Bóta A. Beke-Somfai T. Varga Z. (2017). Characterization of extracellular vesicles by IR spectroscopy: fast and simple classification based on amide and CH stretching vibrations. Biochim. Biophys. Acta 1859, 459–466. 10.1016/j.bbamem.2016.12.005 27989744

[B99] Nawaz M. Shah N. Zanetti B. R. Maugeri M. Silvestre R. N. Fatima F.. (2018). Extracellular vesicles and matrix remodeling enzymes: the emerging roles in extracellular matrix remodeling, progression of diseases and tissue repair. Cells 7:167. 10.3390/cells7100167 30322133 PMC6210724

[B100] Olcum S. Cermak N. Wasserman S. C. Christine K. S. Atsumi H. Payer K. R.. (2014). Weighing nanoparticles in solution at the attogram scale. Proc. Natl. Acad. Sci. U.S.A. 111, 1310–1315. 10.1073/pnas.1318602111 24474753 PMC3910582

[B101] Oliveira-Rodríguez M. López-Cobo S. Reyburn H. T. Costa-García A. López-Martín S. Yá-ez-Mó M.. (2016). Development of a rapid lateral flow immunoassay test for detection of exosomes previously enriched from cell culture medium and body fluids. J. Extracell. Vesicles 5, 2667–2688. 10.3402/jev.v5.31803 27527605 PMC4985618

[B102] Osorio-Querejeta I. Alberro A. Mu-oz-Culla M. Mäger I. Otaegui D. (2018). Therapeutic potential of extracellular vesicles for demyelinating diseases; challenges and opportunities. Front. Mol. Neurosci. 1:434 10.3389/fnmol.2018.00434 PMC6265410 30532691

[B103] Ostrowski M. Carmo N. B. Krumeich S. Fanget I. Raposo G. Savina A.. (2010). Rab27a and Rab27b control different steps of the exosome secretion pathway. Nat. Cell Biol. 12, 19–30. 10.1038/ncb2000 19966785

[B104] Otto A. (2002). What is observed in single molecule SERS, and why? J. Raman Spectr. 33, 593–598. 10.1002/jrs.879

[B105] Park J. Hwang M. Choi B. Jeong H. Jung J.-, h. Kim H. K.. (2017a). Exosome Classification by pattern analysis of surface-enhanced raman spectroscopy data for lung cancer diagnosis. Anal. Chem. 89, 6695–6701. 10.1021/acs.analchem.7b00911 28541032

[B106] Park J. Im H. Hong S. Castro C. M. Weissleder R. Lee H. (2017b). Analyses of intravesicular exosomal proteins using a nano-plasmonic system. ACS Photon. 5, 487–494. 10.1021/acsphotonics.7b00992 29805987 PMC5966285

[B107] Potara M. Gabudean A.-M. Astilean S. (2011). Solution-phase, dual LSPR-SERS plasmonic sensors of high sensitivity and stability based on chitosan-coated anisotropic silver nanoparticles. J. Mater. Chem. 21, 3625–3633. 10.1039/c0jm03329d

[B108] Pugholm L. H. Revenfeld A. L. Søndergaard E. K. Jørgensen M. M. (2015). Antibody-based assays for phenotyping of extracellular vesicles. Biomed Res. Int. 2015, 1–15. 10.1155/2015/524817 26770974 PMC4681819

[B109] Qiu G. Thakur A. Xu C. Ng S.-P. Lee Y. Wu C.-M. L. (2019). Detection of glioma-derived exosomes with the biotinylated antibody-functionalized titanium nitride plasmonic biosensor. Adv. Funct. Mater. 29, 1806761 10.1002/adfm.201806761

[B110] Raghu D. Christodoulides J. A. Christophersen M. Liu J. L. Anderson G. P. Robitaille M.. (2018). Nanoplasmonic pillars engineered for single exosome detection. PLoS ONE 13:202773. 10.1371/journal.pone.0202773 30142169 PMC6108516

[B111] Raposo G. Nijman H. W. Stoorvogel W. Liejendekker R. Harding C. V. Melief C. J.. (1996). B lymphocytes secrete antigen-presenting vesicles. J. Exp. Med. 183, 1161–1172. 10.1084/jem.183.3.1161 8642258 PMC2192324

[B112] Raschke G. Kowarik S. Franzl T. Sönnichsen C. Klar T. A. Feldmann J. (2003). Biomolecular recognition based on single gold nanoparticle light scattering. Nano Lett. 3, 935–938. 10.1021/nl034223+

[B113] Reiner A. T. Fossati S. Dostalek J. (2018). Biosensor platform for parallel surface plasmon-enhanced epifluorescence and surface plasmon resonance detection. Sens. Actuat. Chem. 257, 594–601. 10.1016/j.snb.2017.10.116

[B114] Revenfeld A. L. Bæk R. Nielsen M. H. Stensballe A. Varming K. Jørgensen M. (2014). Diagnostic and prognostic potential of extracellular vesicles in peripheral blood. Clin. Ther. 36, 830–846. 10.1016/j.clinthera.2014.05.008 24952934

[B115] Rezeli M. Gidlöf O. Evander M. Bryl-Górecka P. Sathanoori R. Gilje P. (2016). Comparative proteomic analysis of extracellular vesicles isolated by acoustic trapping or differential centrifugation. Anal. Chem. 88, 8577–8586. 10.1021/acs.analchem.6b01694 27487081

[B116] Ringe E. Langille M. R. Sohn K. Zhang J. Huang J. Mirkin C. A.. (2012). Plasmon Length: a universal parameter to describe size effects in gold nanoparticles. J. Phys. Chem. Lett. 3, 1479–1483. 10.1021/jz300426p 26285624

[B117] Rood I. M. Deegens J. K. Merchant M. L. Tamboer W. P. Wilkey D. W. Wetzels J. F.. (2010). Comparison of three methods for isolation of urinary microvesicles to identify biomarkers of nephrotic syndrome. Kidney Int. 78, 810–816. 10.1038/ki.2010.262 20686450

[B118] Ruach-Nir I. Bendikov T. A. Doron-Mor I. Barkay Z. Vaskevich A. Rubinstein I. (2007). Silica-stabilized gold island films for transmission localized surface plasmon sensing. J. Am. Chem. Soc. 129, 84–92. 10.1021/ja064919f 17199286

[B119] Rupert D. L. Lässer C. Eldh M. Block S. Zhdanov V. P. Lotvall J. O.. (2014). Determination of exosome concentration in solution using surface plasmon resonance spectroscopy. Anal. Chem. 86, 5929–5936. 10.1021/ac500931f 24848946

[B120] Rupert D. L. M. Shelke G. V. Emilsson G. Claudio V. Block S. Lässer C.. (2016). Dual-wavelength surface plasmon resonance for determining the size and concentration of sub-populations of extracellular vesicles. Anal. Chem. 88, 9980–9988. 10.1021/acs.analchem.6b01860 27644331

[B121] Scarabelli L. (2018). Recent advances in the rational synthesis and self-assembly of anisotropic plasmonic nanoparticles. Pure Appl. Chem. 90, 1393–1407. 10.1515/pac-2018-0510

[B122] Sepúlveda B. Angelomé P. C. Lechuga L. M. Liz-Marzán L. M. (2009). LSPR-based nanobiosensors. Nano Today 4, 244–251. 10.1016/j.nantod.2009.04.001

[B123] Shin H. Jeong H. Park J Hong S. Choi Y. (2018). Correlation between cancerous exosomes and protein markers based on surface-enhanced Raman spectroscopy (SERS) and principal component analysis (PCA). ACS Sensors 3, 2637–2643. 10.1021/acssensors.8b01047 30381940

[B124] Shpacovitch V. Hergenröder R. (2018). Optical and surface plasmonic approaches to characterize extracellular vesicles. A review. Analyt. Chim. Acta 1005, 1–15. 10.1016/j.aca.2017.11.066 29389314

[B125] Sina A. A. Vaidyanathan R. Dey S. Carrascosa L. G. Shiddiky M. J. Trau M. (2016). Real time and label free profiling of clinically relevant exosomes. Sci. Rep. 6:30460. 10.1038/srep30460 27464736 PMC4964344

[B126] Sivashanmugan K. Huang W.-L. Lin C.-H. Liao J.-D. Lin C.-C. Su W.-C. (2017). Bimetallic nanoplasmonic gap-mode SERS substrate for lung normal and cancer-derived exosomes detection. J. Taiwan Inst. Chem. Eng. 80, 149–155. 10.1016/j.jtice.2017.09.026

[B127] Skivesen N. Horvath R. Thinggaard S. Larsen N. B. Pedersen H. C. (2007). Deep-probe metal-clad waveguide biosensors. Biosens. Bioelectr. 22, 1282–1288. 10.1016/j.bios.2006.05.025 16828273

[B128] Smith Z. J. Lee C. Rojalin T. Carney R. P. Hazari S. Knudson A.. (2015). Single exosome study reveals subpopulations distributed among cell lines with variability related to membrane content. J. Extracell. Vesicles 4:28533. 10.3402/jev.v4.28533 26649679 PMC4673914

[B129] Sódar B. W. Kittel Á. Pálóczi K. Vukman K. V. Osteikoetxea X. Szabó-Taylor K.. (2016). Low-density lipoprotein mimics blood plasma-derived exosomes and microvesicles during isolation and detection. Sci. Rep. 6:24316. 10.1038/srep24316 27087061 PMC4834552

[B130] Špringer T. Ermini M. L. Špačkov,á B. Jablonk,u J. Homola J. (2014). Enhancing sensitivity of surface plasmon resonance biosensors by functionalized gold nanoparticles: size matters. Anal. Chem. 86, 10350–10356. 10.1021/ac502637u 25226207

[B131] Stewart M. E. Anderton C. R. Thompson L. B. Maria J. Gray S. K Rogers J. A.. (2008). Nanostructured plasmonic sensors. Chem. Rev. 108, 494–521. 10.1021/cr068126n 18229956

[B132] Stremersch S. Marro M. Pinchasik B. E. Baatsen P. Hendrix A. De Smedt S. C.. (2016). Identification of individual exosome-like vesicles by surface enhanced raman spectroscopy. Small 12, 3292–3301. 10.1002/smll.201600393 27171437

[B133] Su J. (2015). Label-free single exosome detection using frequency-locked microtoroid optical resonators. ACS Photonics 2, 1241–1245. 10.1021/acsphotonics.5b00142

[B134] Takov K. Yellon D. M. Davidson S. M. (2017). Confounding factors in vesicle uptake studies using fluorescent lipophilic membrane dyes. J. Extracell. Vesicles 6:1388731. 10.1080/20013078.2017.1388731 29184625 PMC5699187

[B135] Tan A. De La Peña H. Seifalian A. M. (2010). The application of exosomes as a nanoscale cancer vaccine. Int. J. Nanomed. 5, 889–900. 10.2147/IJN.S13402 21116329 PMC2990382

[B136] Tatischeff I. Larquet E. Falcón-Pérez J. M. Turpin P. Y. Kruglik S. G. (2012). Fast characterisation of cell-derived extracellular vesicles by nanoparticles tracking analysis, cryo-electron microscopy, and Raman tweezers microspectroscopy. J. Extracell. Vesicles 1:19179. 10.3402/jev.v1i0.19179 24009887 PMC3760651

[B137] Tauro B. J. Greening D. W. Mathias R. A. Ji H. Mathivanan S. Scott A. M.. (2012). Comparison of ultracentrifugation, density gradient separation, and immunoaffinity capture methods for isolating human colon cancer cell line LIM1863-derived exosomes. Methods 56, 293–304. 10.1016/j.ymeth.2012.01.002 22285593

[B138] Thakur A. Qiu G. Ng S. P. Guan J. Yue J. Lee Y.. (2017). Direct detection of two different tumor-derived extracellular vesicles by SAM-AuNIs LSPR biosensor. Biosens. Bioelectr. 94, 400–407. 10.1016/j.bios.2017.03.036 28324860

[B139] Théry C. Witwer K. W. Aikawa E. Alcaraz M. J. Anderson J. D. Andriantsitohaina R.. (2018). Minimal information for studies of extracellular vesicles 2018 (MISEV2018): a position statement of the international society for extracellular vesicles and update of the MISEV2014 guidelines. J. Extracell. Vesicles 7:1535750. 10.1080/20013078.2018.1535750 30637094 PMC6322352

[B140] Théry C. Zitvogel L. Amigorena S. (2002). Exosomes: composition, biogenesis and function. Nat. Rev. Immunol. 2, 569–579. 10.1038/nri855 12154376

[B141] Tian Y. F. Ning C. F. He F. Yin B. C. Ye B. C. (2018). Highly sensitive detection of exosomes by SERS using gold nanostar@Raman reporter@nanoshell structures modified with a bivalent cholesterol-labeled DNA anchor. Analyst 143, 4915–4922. 10.1039/C8AN01041B 30225507

[B142] Tirinato L. Gentile F. Di Mascolo D. Coluccio M. L. Das G. Liberale C. (2012). SERS analysis on exosomes using super-hydrophobic surfaces. Microelectron. Eng. 97, 337–340. 10.1016/j.mee.2012.03.022

[B143] Tkach M. Kowal J. Théry C. (2018). Why the need and how to approach the functional diversity of extracellular vesicles. Phil. Trans. R. Soc. B. 373:20160479. 10.1098/rstb.2016.0479 29158309 PMC5717434

[B144] Trajkovic K. Hsu C. Chiantia S. Rajendran L Wenzel D. Wieland F.. (2008). Ceramide triggers budding of exosome vesicles into multivesicular endosomes. Science 319, 1244–1247. 10.1126/science.1153124 18309083

[B145] Unser S. Bruzas I. He J. Sagle L. (2015). Localized surface plasmon resonance biosensing: current challenges and approaches. Sensors 15, 15684–15716. 10.3390/s150715684 26147727 PMC4541850

[B146] Vagner T. Spinelli C. Minciacchi V. R. Balaj L. Zandian M. Conley A.. (2018). Large extracellular vesicles carry most of the tumour DNA circulating in prostate cancer patient plasma. J. Extracell. Vesicles 7:1505403. 10.1080/20013078.2018.1505403 30108686 PMC6084494

[B147] Valadi H. Ekström K. Bossios A. Sjöstrand M. Lee J. J. Lötvall J. O. (2007). Exosome-mediated transfer of mRNAs and microRNAs is a novel mechanism of genetic exchange between cells. Nat. Cell Biol. 9, 654–659. 10.1038/ncb1596 17486113

[B148] van der Pol E. de Rond L. Coumans F. A. W. Gool E. L. Böing A. N. Sturk A.. (2018). Absolute sizing and label-free identification of extracellular vesicles by flow cytometry. Nanomed. Nanotechnol. Biol. Med. 14, 801–810. 10.1016/j.nano.2017.12.012 29307842

[B149] van der Vlist E. J. Nolte-'t Hoen E. N. Stoorvogel W. Arkesteijn G. J. Wauben M. H. (2012). Fluorescent labeling of nano-sized vesicles released by cells and subsequent quantitative and qualitative analysis by high-resolution flow cytometry. Nat. Protoc. 7, 1311–1326. 10.1038/nprot.2012.065 22722367

[B150] Vestad B. Llorente A. Neurauter A. Phuyal S. Kierulf B. Kierulf P.. (2017). Size and concentration analyses of extracellular vesicles by nanoparticle tracking analysis: a variation study. J. Extracell. Vesicles 6:1344087. 10.1080/20013078.2017.1344087 28804597 PMC5533132

[B151] Wang Z. Zong S. Wang Y. Li N. Li L Lu J. (2018). Screening and multiple detection of cancerous exosomes using a SERS-based method. Nanoscale 10, 9053–9062. 10.1039/C7NR09162A 29718044

[B152] Webber J. Clayton A. (2013). How pure are your vesicles? J. Extracell. Vesicles 2, 19861. 10.3402/jev.v2i0.19861 24009896 PMC3760653

[B153] Wei Z. Batagov A. O. Schinelli S. Wang J. Wang Y. El Fatimy R.. (2017). Coding and noncoding landscape of extracellular RNA released by human glioma stem cells. Nat. Commun. 8:1145. 10.1038/s41467-017-01196-x 29074968 PMC5658400

[B154] Willets K. A. Van Duyne R. P. (2007). Localized surface plasmon resonance spectroscopy and sensing. Annu. Rev. Phys. Chem. 58, 267–297. 10.1146/annurev.physchem.58.032806.104607 17067281

[B155] Williams C. Royo F. Aizpurua-Olaizola O. Pazos R. Boons G. J. Reichardt N. C.. (2018). Glycosylation of extracellular vesicles: current knowledge, tools and clinical perspectives. J. Extracell. Vesic. 7:1442985. 10.1080/20013078.2018.1442985 29535851 PMC5844028

[B156] Wilson B. C. Jermyn M. Leblond F. (2018). Challenges and opportunities in clinical translation of biomedical optical spectroscopy and imaging. J. Biomed. Opt. 23:030901. 10.1117/1.JBO.23.3.030901 29512358 PMC5838403

[B157] Witwer K. W. Buzás E. I. Bemis L. T. Bora A. Lässer C. Lötvall J.. (2013). Standardization of sample collection, isolation and analysis methods in extracellular vesicle research. J. Extracell. Vesicles 2:20360. 10.3402/jev.v2i0.20360 24009894 PMC3760646

[B158] Witwer K. W. Soekmadji C. Hill A. F. Wauben M. H. Buzás E. I. Di Vizio D.. (2017). Updating the MISEV minimal requirements for extracellular vesicle studies: building bridges to reproducibility. J. Extracell. Vesicles 6:1396823. 10.1080/20013078.2017.1396823 29184626 PMC5698937

[B159] Wunsch B. H. Smith J. T. Gifford S. M. Wang C. Brink M. Bruce R.. (2016). Nanoscale lateral displacement arrays for the separation of exosomes and colloids down to 20 nm. Nat. Nanotechnol. 11, 936–940. 10.1038/nnano.2016.134 27479757

[B160] Xi Z. Ye H. Xia X. (2018). Engineered noble-metal nanostructures for *in vitro* diagnostics. Chem. Mater. 30, 8391–8414. 10.1021/acs.chemmater.8b04152

[B161] Xia F. Zuo X. Yang R. Xiao Y. Kang D. Vallée-Bélisle A.. (2010). Colorimetric detection of DNA, small molecules, proteins, and ions using unmodified gold nanoparticles and conjugated polyelectrolytes. Proc. Natl. Acad. Sci. U.S.A. 107, 10837–10841. 10.1073/pnas.1005632107 20534499 PMC2890709

[B162] Xu R. Simpson R. J. Greening D. W. (2016). A Protocol for Isolation and Proteomic Characterization of Distinct Extracellular Vesicle Subtypes by Sequential Centrifugal Ultrafiltration. Methods in Molecular Biology. New York, NY: Springer New York. 10.1007/978-1-4939-6728-5_7 27943209

[B163] Yáñez-Mó M. Siljander P. R. Andreu Z. Zavec A. B. Borràs F. E. Buzas E. I.. (2015). Biological properties of extracellular vesicles and their physiological functions. J. Extracell. Vesicles 4:27066. 10.3402/jev.v4.27066 25979354 PMC4433489

[B164] Yang K. S. Im H. Hong S. Pergolini I. Del Castillo A. F. Wang R.. (2017). Multiparametric plasma EV profiling facilitates diagnosis of pancreatic malignancy. Sci. Transl. Med. 9:391. 10.1126/scitranslmed.aal3226 28539469 PMC5846089

[B165] Yang X. Zhao X. Yang K. Liu Y. Liu Y. Fu W.. (2016). Biomedical applications of terahertz spectroscopy and imaging. Trends Biotechnol. 34, 810–824. 10.1016/j.tibtech.2016.04.008 27207226

[B166] Yang Y. Shen G. Wang H. Li H. Zhang T. Tao N.. (2018). Interferometric plasmonic imaging and detection of single exosomes. Proc. Natl. Acad. Sci. U.S.A. 115, 10275–10280. 10.1073/pnas.1804548115 30249664 PMC6187158

[B167] Zeng X. Yang Y. Zhang N. Ji D. Gu X. Journet J. M.. (2019). Plasmonic interferometer array biochip as a new mobile medical device for cancer detection. IEEE J. Selected Top. Quantum Electr. 25, 1–7. 10.1109/JSTQE.2018.2865418 30983848 PMC6456910

[B168] Zhang H. Freitas D. Kim H. S. Fabijanic K. Li Z. Chen H.. (2018). Identification of distinct nanoparticles and subsets of extracellular vesicles by asymmetric flow field-flow fractionation. Nat. Cell Biol. 20, 332–343. 10.1038/s41556-018-0040-4 29459780 PMC5931706

[B169] Zhang P. He M. Zeng Y. (2016). Ultrasensitive microfluidic analysis of circulating exosomes using a nanostructured graphene oxide/polydopamine coating. Lab Chip 16, 3033–3042. 10.1039/C6LC00279J 27045543 PMC4970962

[B170] Zhang Y. Wang G. Yang L. Wang F. Liu A. (2018). Recent advances in gold nanostructures based biosensing and bioimaging. Coord. Chem. Rev. 370, 1–21. 10.1016/j.ccr.2018.05.005

[B171] Zhao X. Huang T. Ping P. S. Wu X. Huang P. Pan J.. (2018). Sensitivity enhancement in surface plasmon resonance biochemical sensor based on transition metal dichalcogenides/graphene heterostructure. Sensors 18:2056. 10.3390/s18072056 29954134 PMC6068701

[B172] Zheng X. S. Jahn I. J. Weber K. Cialla-May D. Popp J. (2018). Label-free SERS in biological and biomedical applications: recent progress, current challenges and opportunities. Spectrochim. Acta Part A Mol. Biomol. Spectrosc. 197, 56–77. 10.1016/j.saa.2018.01.063 29395932

[B173] Zhu J. Qin L. Song S. Zhong J. Lin S. (2015). Design of a surface plasmon resonance sensor based on grating connection. Photonic Sensors 5, 159–165. 10.1007/s13320-015-0244-1

[B174] Zhu L. Wang K. Cui J. Liu H. Bu X. Ma H.. (2014). Label-free quantitative detection of tumor-derived exosomes through surface plasmon resonance imaging. Anal. Chem. 86, 8857–8864. 10.1021/ac5023056 25090139 PMC4151789

[B175] Zhu S. Li H. Yang M. Pang S. W. (2018). Highly sensitive detection of exosomes by 3D plasmonic photonic crystal biosensor. Nanoscale 10, 19927–19936. 10.1039/C8NR07051B 30346006

[B176] Zijlstra A. Di Vizio D. (2018). Size matters in nanoscale communication. Nat. Cell Biol. 20, 228–230. 10.1038/s41556-018-0049-8 29476154 PMC6652179

[B177] Zong S. Wang L. Chen C. Lu J. Zhu D. Zhang Y. (2016). Facile detection of tumor-derived exosomes using magnetic nanobeads and SERS nanoprobes. Anal. Methods 8, 5001–5008. 10.1039/C6AY00406G

